# Kidney Organoid Modeling of WT1 Mutations Reveals Key Regulatory Paths Underlying Podocyte Development

**DOI:** 10.1002/advs.202308556

**Published:** 2024-05-29

**Authors:** Gang Wang, Hangdi Wu, Xiuwen Zhai, Li Zhang, Changming Zhang, Chen Cheng, Xiaodong Xu, Erzhi Gao, Xushen Xiong, Jin Zhang, Zhihong Liu

**Affiliations:** ^1^ National Clinical Research Center of Kidney Diseases Jinling Hospital Nanjing University School of Medicine Nanjing Jiangsu 210002 China; ^2^ Department of Basic Medical Sciences Zhejiang University School of Medicine Hangzhou Zhejiang 310058 China; ^3^ Liangzhu Laboratory Zhejiang University Hangzhou 311121 China; ^4^ Center for Stem Cell and Regenerative Medicine Department of Basic Medical Sciences & The First Affiliated Hospital Zhejiang University School of Medicine Hangzhou Zhejiang 310058 China; ^5^ State Key Laboratory of Transvascular Implantation Devices The Second Affiliated Hospital Zhejiang University School of Medicine Hangzhou 311121 China; ^6^ Hematology Institute Zhejiang University Hangzhou Zhejiang 310058 China

**Keywords:** kidney organoid, podocyte development, scATAC‐seq, scRNA‐seq, WT1

## Abstract

Wilms tumor‐1(WT1) is a crucial transcription factor that regulates podocyte development. However, the epigenomic mechanism underlying the function of WT1 during podocyte development has yet to be fully elucidated. Here, single‐cell chromatin accessibility and gene expression maps of foetal kidneys and kidney organoids are generated. Functional implications of WT1‐targeted genes, which are crucial for the development of podocytes and the maintenance of their structure, including *BMPER*/*PAX2/MAGI2* that regulates WNT signaling pathway, *MYH9* that maintains actin filament organization and *NPHS1* that modulates cell junction assembly are identified. To further illustrate the functional importance of WT1‐mediated transcriptional regulation during podocyte development, cultured and implanted patient‐derived kidney organoids derived from the Induced Pluripotent Stem Cell (iPSCs) of a patient with a heterozygous missense mutation in *WT1* are generated. Results from single‐cell RNA sequencing (scRNA‐seq) and functional assays confirm that the *WT1* mutation leads to delays in podocyte development and causes damage to cell structures, due to its failure to activate the targeting genes *MAGI2, MYH9*, and *NPHS1*. Notably, correcting the mutation in the patient iPSCs using CRISPR‐Cas9 gene editing rescues the podocyte phenotype. Collectively, this work elucidates the WT1‐related epigenomic landscape with respect to human podocyte development and identifies the disease‐causing role of a *WT1* mutation.

## Graphical abstract

In the present study, the WT1‐related epigenomic landscape of human podocyte development is elucidated by single‐cell multiomic dissection of foetal kidney and kidney organoids, revealing a substantial epigenomic similarity between kidney organoids and their foetal kidney counterparts. Furthermore, cultured and implanted kidney organoids desirably model the podocyte phenotypes and reveal the disease‐causing role and the underlying mechanism of a newly identified *WT1* mutation.

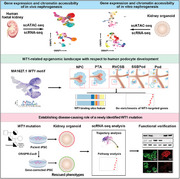

## 1 Introduction

During kidney development, *WT1* expression is increased along with the development of metanephric mesenchyme and podocyte progenitors, and it is ultimately restricted to mature podocytes.^[^ ^1^ ^]^ WT1 acts as a crucial transcription factor that regulates podocyte development and maintains podocyte structure and function.^[^ ^2^ ^]^ Previous studies have profiled the WT1‐related epigenomic landscape of developing and damaged mouse kidneys via WT1 ChIP(chromatin immunoprecipitation) assays, thereby greatly enhancing our understanding of WT1 binding genes, which are important for podocyte development and function.^[^ ^3^, ^4^ ^]^ However, a comprehensive investigation of the cell type‐resolved WT1‐related epigenomic landscape across differentiation trajectories in human podocyte development is lacking.

Foetal kidney samples are extremely scarce, making it difficult to use them for a thorough WT1‐ChIP experiment. With the development of single‐cell ATAC sequencing (scATAC‐seq) and scRNA‐seq technology, the above challenge can be effectively solved. Integrative single‐cell analysis can investigate the role of all transcription factors at a cell‐type‐resolved level.^[^ ^5^ ^]^ In addition, previous studies have shown a certain degree of similarity between kidney organoids and foetal kidneys.^[^ ^6^ ^]^ Moreover, kidney organoid is a well‐established disease model for exploring the pathogenic mechanism of patient gene mutations.^[^ ^7^, ^8^ ^]^ Therefore, combining foetal kidney and kidney organoid with single‐cell analysis provides an opportunity to comprehensively elucidate the role of WT1 in podocyte development.

Mutations in *WT1* result in various podocyte manifestations, which are accompanied by a defective glomerular basement membrane.^[^ ^9^ ^]^ To understand how *WT1* mutations lead to a dysregulated podocyte phenotype, multiple animal models have been generated and characterized. For instance, in a previous study, WT1 conditional knockout mice were generated to elucidate WT1 function in podocytes during the time course of development.^[^ ^4^ ^]^ However, previous studies on how *WT1* mutations lead to the molecular and cellular phenotype simply focused on the role of a single gene or events that occurred at a specific developmental time point rather than at a systematic level. As mentioned above, WT1 plays a role throughout the whole process of podocyte maturation and affects the function of multiple downstream target genes. Therefore, to systematically describe the molecular and cellular phenotypes of *WT1* mutations, we adopted cultured and implanted kidney organoids that preserve the genetic background of patients, which dynamically recapitulates the molecular and cellular phenotype of podocyte damage induced by *WT1* mutation.

Here, we established single‐cell chromatin accessibility and gene expression maps of foetal kidney and kidney organoids by scATAC‐seq and scRNA‐seq experiments. We first profiled the WT1‐related epigenomic landscape of podocyte development spanning NPC/nephron progenitor cells, PTA/pretubular aggregates, SSBPod/s‐shaped body podocyte precursor cells, and podocytes by analyzing the distribution of the WT1 motif. We determined the number and position of the WT1 binding motifs in the four cell types mentioned above. We then identified the functional implications of WT1‐targeted genes, which are crucial for the podocyte development and the maintenance of podocyte structure, such as the WNT signaling pathway (*BMPER/PAX2/MAGI2*), actin filament organization (*MYH9*) and cell junction assembly (*NPHS1*). In addition, we performed a systematic comparison between the WT1‐related epigenome atlas of foetal podocyte development and kidney organoids. The comparison of NPC/SSBPod/podocyte cells of foetal kidney and kidney organoids confirmed that the WT1‐related developmental trajectory of kidney organoids shows substantial epigenomic similarity to foetal kidney counterparts. Finally, to confirm the importance of WT1‐mediated transcriptional function during podocyte development, we generated cultured and implanted patient‐derived kidney organoids using a nephrotic syndrome patient‐iPSCs that carried a heterozygous missense mutation in *WT1* (c.1306A > G, exon 8). ScRNA‐seq analysis and functional assays revealed that the *WT1* mutation caused podocyte development delays and cell structure damage due to the down‐regulation of genes involved in the WNT signaling pathway (*MAGI2*), actin filament organization (*MYH9*) and cell junction assembly (*NPHS1*). Notably, correcting the mutation in patient iPSCs using CRISPR–Cas9 gene editing enables the organoids to rescue the podocyte phenotype. Together, we define the WT1‐related epigenomic landscape of human podocyte development, reveal that kidney organoids have substantial epigenomic similarity to their foetal kidney counterparts, and identify the disease‐causing role of the newly identified *WT1* mutation.

## 2 Results

### 2.1 Determining the WT1‐Related Epigenomic Landscape of Human Podocyte Development Across Major Cellular Differentiation Trajectories in Foetal Kidneys

**Figure 1:**
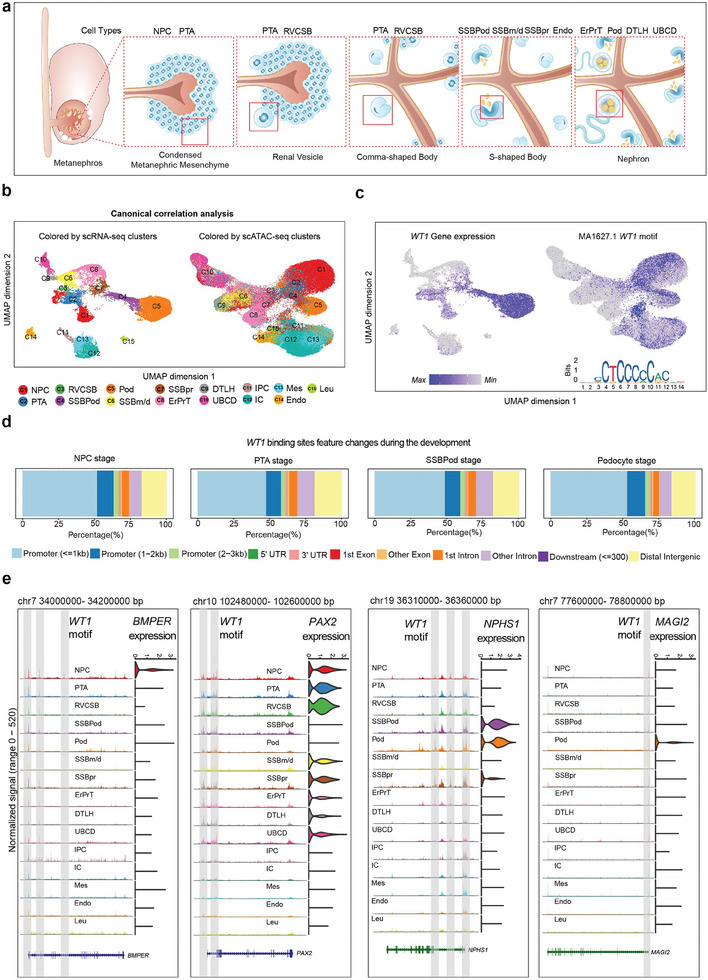
Dynamics of WT1 binding during foetal kidney development. a) The various stages of nephron development are shown. The sequence of developmental steps: metanephric mesenchyme, renal vesicle, comma‐shaped bodies, S‐shaped body, nephron. The part of the *WT1* expression is depicted in the red box. Cell types in the stages of nephron development: NPC/nephron progenitor cell; PTA/pretubular aggregate; RVCSB/renal vesicle/comma‐shaped body; SSBPod/s‐shaped body podocyte precursor cells; SSBm/d/s‐shaped body medial/distal; SSBpr/s‐shaped body proximal precursor cells; Endo/endothelial cells; pod/podocytes; ErPrT/early proximal tubule; DTLH/distal tubule/loop of Henle; UBCD/ureteral bud/collecting duct. b) UMAPs of scRNA‐seq and scATAC‐seq cells colored by cluster assignment in their respective data modality and UMAP of scATAC‐seq cells highlighted by complementary scRNA‐seq clusters. C1, NPC/nephron progenitor cell; C2, PTA/pretubular aggregate; C3, RVCSB/renal vesicle/comma‐shaped body; C4, SSBPod/s‐shaped body podocyte precursor cells; C5, pod/podocytes; C6, SSBm/d/s‐shaped body medial/distal; C7, SSBpr/s‐shaped body proximal precursor cells; C8, ErPrT/early proximal tubule; C9, DTLH/distal tubule/loop of Henle; C10, UBCD/ureteral bud/collecting duct; C11, IPC/interstitial progenitor cells; C12, IC/interstitial cells; C13, Mes/mesangial cells; C14, Endo/endothelial cells; C15, Leu/leukocytes. c) scRNA‐seq‐based mRNA expression of *WT1* in the scRNA‐seq UMAP representations of all cells. scATAC‐seq‐based ChromVAR motif deviation scores for *WT1* are shown in the scATAC‐seq UMAP representations of all cells. d) Genomic distribution of all WT1 binding sites in the NPC, PTA, SSBPod, and Podocyte stage. e) Left column: Genome tracks of cell type‐resolved aggregate scATAC‐seq data around the *BMPER*, *PAX2*, *NPHS1*, and *MAGI2* gene loci. Right column: Distribution of scRNA‐seq gene expression of *BMPER*, *PAX2*, *NPHS1*, and *MAGI2* across cell type clusters.

To capture chromatin dynamics data in different cell populations throughout foetal kidney development (**Figure** **1**a), we used scATAC‐seq data from a previous study that profiled six primary human foetal kidney samples at 13, 16, 17, and 18 weeks after postconception.^[^ ^10^ ^]^ To establish the regulation between the chromatin and gene expression landscapes of cell types, we integrated a previously published scRNA‐seq dataset from developmental time points of 9, 11, 13, 15, 16, and 18 weeks after postconception.^[^ ^11^ ^]^ The scATAC‐seq atlas were clustered into 15 cell types, which matched with their nearest neighbor cells in the scRNA‐seq atlas using canonical correlation analysis (Figure 1b; Figure S1a, Supporting Information).

To elucidate the WT1‐related epigenomic landscape during human podocyte development, we examined *WT1* mRNA expression and its motif open activity score (chromVAR) by using the multiomic atlas integrated above. The WT1 motif open activity score and the expression of *WT1* were specific in the NPC, PTA, SSBPod, and podocyte clusters, which were corroborated with high concordance (Figure 1c; Figure S1b, Supporting Information). The NPC‐PTA‐SSBPod‐podocyte developmental trajectory is the maturation process of podocytes in vivo. These results indicated the important role of WT1 during podocyte development.

Furthermore, we determined the number and distribution of WT1 motifs located in the peaks in the four cell types mentioned above. The global distribution of WT1 binding sites did not change during podocyte development, which was mostly located at gene promoters and putative distal enhancers (Figure 1d). Thus, WT1 binding is a major determinant of gene expression in podocyte development, and binding at the promoter region is particularly important. Besides, we also compared the adjacent developmental stages of genomic WT1 binding sites from NPC to Podocyte (Figure S1c,d, Supporting Information). In early kidney development (NPC and PTA stages), the global number of WT1 binding sites exceeds 9000 (Figure S1c, Supporting Information). As podocyte development proceeded (SSBPod and podocyte stages), the global number of WT1 binding sites was greatly reduced (Figure S1c, Supporting Information). These results demonstrate a process whereby WT1 acquires a substantially increased binding site during the early stages of kidney development to endow progenitor cells with multidirectional differentiation characteristics. As development progresses, the main function of WT1 is to maintain the identity and function of podocytes, therefore the global number of WT1 binding sites maintains at a stable level compared to the early stage.

Next, we focused on NPC/PTA/SSBpod/podocytes to examine the functional implications of WT1‐targeted genes. Gene Ontology (GO) analysis‐based scATAC‐seq datasets revealed enrichments in the pathways of kidney development, WNT signaling pathway, and epithelial tube morphogenesis at the NPC/PTA/SSBPod stages (Figure S2, arrow indication, Supporting Information). In addition, GO analysis indicated that cell junction assembly and actin filament organization pathways were enriched at the podocyte stage (Figure S2, arrow indication, Supporting Information). The WNT signaling pathway plays an important role in maintaining the proliferation and differentiation of nephron progenitor cells, as well as regulating the MET pathway.^[^ ^1^ ^]^ The WNT signaling pathway molecules *BMPER* and *PAX2 were* specifically expressed in the NPC and PTA cluster, and WT1 motifs were also found in the promoter and enhancer region of these two genes (Figure 1e). The cell junction assembly and actin filament organization could regulate and maintain the specific foot‐like structure of podocytes.^[^ ^2^ ^]^ Our results showed that the cell junction assembly genes, *NPHS1* and *MAGI2*, were specifically expressed in the podocyte cluster and that WT1 motifs were also located in the promoter and enhancer region of these two genes (Figure 1e). In addition, the WNT signaling pathway also plays an important role during kidney organoid induction.^[^ ^12^, ^13^ ^]^ Collectively, we determined the WT1‐related epigenomic landscape of human podocyte development based on integrative single‐cell analysis of foetal kidneys.

To verify the feasibility of using scATAC‐seq to analyze the positions of the WT1 motif and to predict the WT1 binding sites, we integrated previously a published WT1 ChIP‐seq dataset that focused on E18.5 kidneys for comparison and confirmation.^[^ ^3^ ^]^ Our scATAC‐seq analysis showed that the *WT1* expression and motif open activity score in the NPC, PTA, RVCSB, SSBPod, and podocyte clusters were significantly increased (Figure 1c; Figure S1b, Supporting Information). We merged these five sub‐clusters and renamed them as the WT1 cluster (Figure S3a,b, Supporting Information). The WT1 cluster is the main cell type that undergoes a binding assay in the WT1 ChIP experiment of mouse kidneys. The global distribution of WT1 binding sites between the 2 datasets was similar, which suggests that WT1 may predominantly function through distal regulatory elements and promoter regions (Figure S3d,e, Supporting Information). We then compared the genes around the WT1 motif between mouse kidney ChIP‐Seq (10222 genes) and human kidney scATAC‐seq (9871 genes) and found that 52% of these genes were overlapped (Figure S3c, Supporting Information). The overlapping genes included *MAGI2*, *MYH9*, *ITGA3*, *SYNPO*, and *WT1*, which were known to maintain the identity and function of podocytes (Figure S3c, Supporting Information). We next examined the functional implications of the WT1‐targeted genes. GO analysis based on the two datasets was highly consistent with each other, showing robust enrichments in the WNT signaling pathway, actin filament organization, renal system development, and regulation of GTPase activity (Figure S3f,g, arrow indication, Supporting Information). Therefore, we reliably revealed the distribution and binding targets of WT1 using scATAC‐seq.

### 2.2 Determining the WT1‐Related Epigenomic Landscape of Human Podocyte Development Across Major Cellular Differentiation Trajectories in Cultured Kidney Organoids

Recent studies have shown that kidney organoids generated by iPSCs derived from patients with inherited kidney diseases manifest a glomerular phenotype.^[^ ^7^, ^8^ ^]^ Therefore, confirming the similarity between kidney and kidney organoids is important. Previous studies that profiled the scRNA‐seq of foetal kidney and kidney organoids have greatly enhanced our understanding of the transcriptomic landscape across cell types, which indicated that foetal kidney and kidney organoids are similar to each other at the transcriptomic level.^[^ ^6^ ^]^ However, a comprehensive resource of cell type‐resolved cis and trans regulators of gene expression programs across differentiation trajectories based on foetal kidney and kidney organoids is lacking. WT1 acts as a crucial transcription factor that regulates podocyte development and maintains podocyte structure and function. Thus, we established the WT1‐related epigenomic landscape of human podocyte development to interrogate the similarity between kidney and kidney organoids. The cell types at key time points during cultured kidney organoid differentiation were confirmed (Figure S4a, Supporting Information).

**Figure 2:**
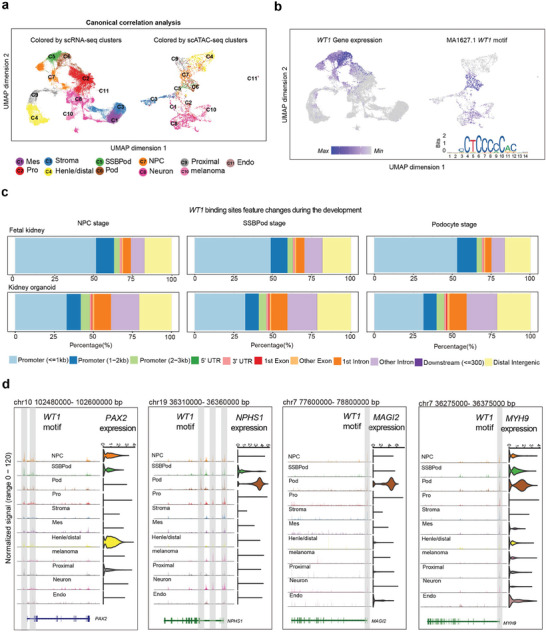
Dynamics of WT1 binding during development in cultured kidney organoids. a) UMAPs of scRNA‐seq and scATAC‐seq cells colored by cluster assignment in their respective data modality and UMAP of scATAC‐seq cells highlighted by complementary scRNA‐seq clusters. C1, Mes/mesangial cells; C2, Pro/proliferating cells; C3, stroma; C4, distal tubule/loop of Henle; C5, SSBPod/S‐shaped body podocyte precursor cells; C6, Pod/podocytes; C7, NPC/nephron progenitor cell; C8, neuron; C9, proximal/proximal tubule; C10, melanoma; C11, Endo/endothelial cells. b) scRNA‐seq‐based mRNA expression of *WT1* in the scRNA‐seq UMAP representations of all cells. scATAC‐seq‐based ChromVAR motif deviation scores for *WT1* are shown in the scATAC‐seq UMAP representations of all cells. c) Genomic distribution of all WT1 binding sites in the NPC, SSBPod, and Podocyte stage. d) Left column: Genome tracks of cell type‐resolved aggregate scATAC‐seq data around the *PAX2*, *NPHS1*, *MAGI2*, and *MYH9* gene loci. Right column: Distribution of scRNA‐seq gene expression of *PAX2*, *NPHS1*, *MAGI2*, and *MYH9* across cell type clusters.

We generated scATAC‐seq and scRNA‐seq data from cultured kidney organoids. To elucidate the correspondence between the chromatin and gene expression landscapes of cell types, we analyzed an integrative dataset of cultured kidney organoids. The scATAC‐seq atlas was divided into 11 cell types, which matched with their nearest neighbor cells in the scRNA‐seq atlas using canonical correlation analysis (**Figure** **2**a). The WT1 motif open activity score and the expression of *WT1* were specific in the NPC, SSBPod, and podocyte clusters, which were corroborated with high concordance (Figure 2b; Figure S6a, Supporting Information). The global distribution of WT1 binding sites showed a negligible change during podocyte development of kidney organoids, which were mostly located at gene promoters and putative distal enhancers, similar to the foetal kidney (Figure 2c). Next, we compared the functional implications of WT1‐targeted genes between foetal kidney and kidney organoid (Figure S5, arrow indication, Supporting Information). Gene Ontology (GO) analysis revealed similar pathway enrichments in the WNT signaling pathway and epithelial tube morphogenesis in the NPC/SSBPod stages, actin filament organization, and cell junction assembly in the podocyte stage (Figure S5, arrow indication, Supporting Information). The WNT signaling pathway molecule *PAX2* was specifically expressed in the NPC/SSBPod cluster, and the WT1 motif was located in the *PAX2* promoter and enhancer regions (Figure 2d). The cell junction assembly and actin filament organization could regulate and maintain the specific foot‐like structure of podocytes. The cell junction assembly molecules *NPHS1*, *MAGI2*, and *MYH9* were specifically expressed in the podocyte cluster, with WT1 motifs found in their promoter and enhancer regions (Figure 2d).

In addition, we also conducted a combined scATAC‐seq analysis of implanted and cultured kidney organoids. However, the scATAC‐seq clustering result was not desirable, due to the mixed mouse cells in the implanted kidney organoids. There was obvious interference with a cell cluster (Figure S6b, Supporting Information). Nevertheless, *WT1* also exhibited strong accessibility at their promoters, and their expression was also specifically high in the SSBPod and podocyte clusters (Figure S6c, Supporting Information).

Overall, we elucidated the WT1‐related epigenomic landscape of human podocyte development based on the integrative single‐cell analysis of kidney organoids. These analyses also confirm the similarity between kidneys and kidney organoids.

### 2.3 Dysregulated Cellular Processes in the SSBPod and Podocytes of Cultured Kidney Organoids Induced by *WT1* Mutations

To confirm the role of WT1 in podocyte development, we generated a WT1‐Knock‐out (KO) iPS cell line. The KO mutation is located in exon 8, which encodes a zinc finger structure (Figure S7c, Supporting Information). Mutations in exons 8 and 9 of *WT1* usually lead to abnormal zinc finger structures that disrupt DNA‐binding activity, which may cause a variety of inherited kidney diseases, including nephrotic syndrome, Wilms tumor, DDS syndrome, and so on.^[^ ^14^, ^15^ ^]^ Upon differentiation, the WT1‐KO kidney organoids exhibited no nephron‐like structures and consisted almost exclusively of stromal cells (Figure S7a,b, Supporting Information). These results confirmed the important role of WT1 in kidney development.

**Figure 3:**
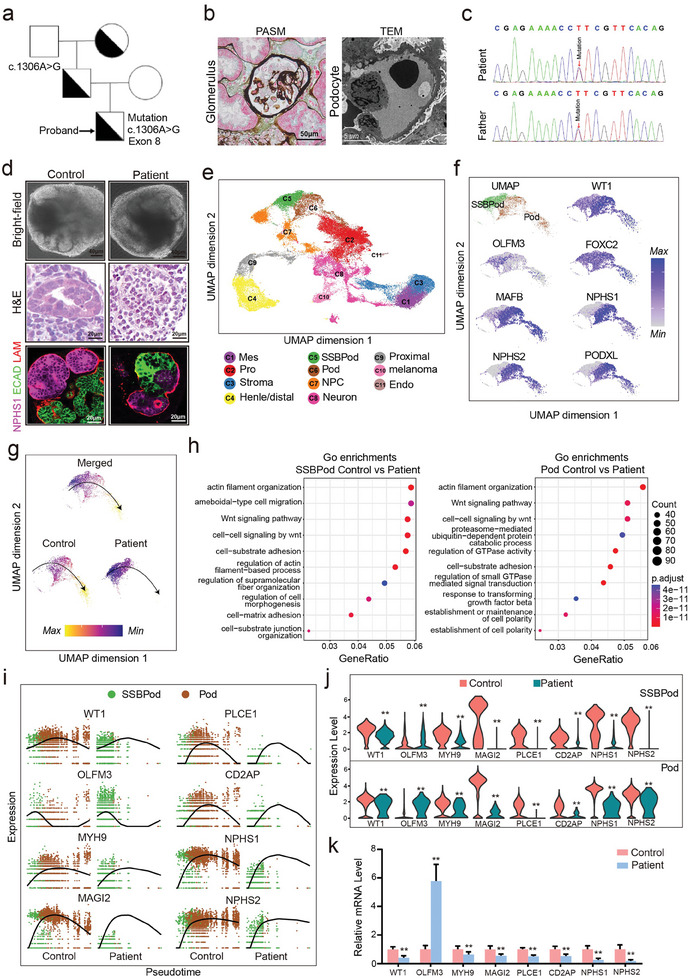
ScRNA‐seq reveals a gene expression profile defect in podocytes from cultured kidney organoids from a patient. a) Pedigrees of the proband and the WT1 variant at c.1306A>G (p.R436G). b) PASM staining and transmission electron microscope assays showed focal segmental glomerulosclerosis of glomerulus and fusion of the foot process of podocyte. c) Sanger sequencing (3′→5′) of the proband and his father. d) Bright‐field images of control and patient‐cultured kidney organoids on day 25.H&E staining shows that control and patient‐cultured kidney organoids contain glomerular structures. Confocal immunofluorescence images display that control and patient‐cultured kidney organoids contain nephron compartments, podocytes (NPHS1+), and tubules (ECAD+), basement membrane (LAM+). e) uMAP plot depicting ten major cell clusters in cultured kidney organoids of both groups (control group *n* = 3, patient group *n* = 2) on day 25. C1, Mes/mesangial cells; C2, Pro/proliferating cells; C3, stroma; C4, distal tubule/loop of Henle; C5, SSBPod/S‐shaped body podocyte precursor cells; C6, Pod/podocytes; C7, NPC/nephron progenitor cell; C8, neuron; C9, proximal/proximal tubule; C10, melanoma; C11, Endo/endothelial cells. f) scRNA‐seq of WT1, OLFM3, FOXC2, MAFB, NPHS1, NPHS2 and PODXL in the SSBpod and podocyte clusters. g) Pseudotime analysis showing the developmental relationship of SSBpod and podocyte clusters. h) GO functional enrichment analysis with differentially expressed genes in the SSBpod and podocyte clusters. The log‐transformed *p*‐value was used to measure significance, and the significance cutoff was set at *p* < 0.05. i) Expression of the WT1, PLCE1, OLFM3, CD2AP, MYH9, NPHS1, MAGI2 and NPHS2 genes across the pseudotime trajectory colored by cell type. j) Distribution of scRNA‐seq gene expression of the *WT1*, *PLCE1*, *OLFM3*, *CD2AP*, *MYH9*, *NPHS1*, *MAGI2*, and *NPHS2* genes in the SSBpod and podocyte clusters. Data are presented as the mean ± SEM, *p < 0.05 and **p < 0.01 between groups. k) Relative mRNA levels of the *WT1*, *OLFM3*, *MYH9*, *MAGI2*, *CD2AP*, *NPHS1*, and *NPHS2* genes in control and patient‐cultured kidney organoids (*n* = 6 per group). Data are presented as the mean ± SEM, *p < 0.05 and **p < 0.01 between groups.

Next, we generated cultured kidney organoids derived from patient iPSCs carrying a *WT1* mutation to model the podocyte phenotype. The patient was diagnosed with nephrotic syndrome at 13 years old, and the mutation originated from his father (**Figure** **3**a). His father has received kidney transplant treatment for ESRD, and his grandmother also receives dialysis treatment for ESRD. PASM staining and transmission electron microscope assays showed focal segmental glomerulosclerosis and fusion of the foot process of podocyte (Figure 3b). The sequencing analysis identified a heterozygous, single‐nucleotide WT1 variant at c.1306A > G (exon8, p.R436G, NM_024426.6) in this patient (Figure 3c). The patient presents with typical clinical symptoms of nephrotic syndrome, such as excessive proteinuria and hyperlipidemia (Figure S7d, Supporting Information). The site is predicted to be damaged by PolyPhen(Figure S7e, Supporting Information) and highly conserved throughout the across species(Figure S7f, Supporting Information). Upon differentiation, both the control and patient‐derived kidney organoids exhibited nephron‐like structures containing podocytes (Figure 3d). Kidney organoids were further validated by immunofluorescence staining of NPHS1 (staining podocytes), ECAD (staining kidney tubule), and LAM (staining basement membrane) (Figure 3d). Since scRNA‐seq can provide unprecedented insights into normal and abnormal kidney cell types,^[^ ^16^ ^]^ we next performed scRNA‐seq to dissect the cellular composition in control and patient‐derived kidney organoids (Figure 3e). We first performed scRNA‐seq for both control (*n* = 3) and patient (*n* = 2) cultured kidney organoids on day 25 after organoid differentiation. We identified 11 major cell clusters based on the expression of known marker genes (Figure 3e, Figure S7g,h, Supporting Information).

Furthermore, we focused on the SSBPod and podocyte cell clusters to investigate the molecular mechanism underlying the podocyte phenotype caused by the *WT1* mutation. Both clusters showed high *WT1* gene expression (Figure 3f). Cluster SSBPod showed high expression of the corresponding marker genes *OLFM3* and *FOXC2*, whereas cluster podocyte showed high expression of its markers, including *MAFB*, *NPHS1*, *NPHS2*, and *PODXL* (Figure 3f). In addition, pseudotime analysis showed that the differentiation time point of the podocyte clusters lagged behind the SSBpod cluster (Figure 3g). The two clusters were represented in both control and patient datasets, but the proportion of cells varied dramatically. Specifically, SSBPod cells made up a large proportion of the patient sample, while podocytes made up a large proportion of the control sample (Figure 3g). These results indicated that the podocyte developmental process was delayed in the patient‐derived kidney organoid. Moreover, GO analysis of the two clusters confirmed the roles of this *WT1* mutation in podocyte development and structural pathways, such as the WNT signaling pathway, actin filament organization, and cell‐matrix adhesion (Figure 3h). Gene expression along the pseudotime trajectory and across cell clusters shows an expected change of key pathway marker genes (Figure 3i). Differential gene expression analysis showed gene expression defects in podocytes and SSBPods in the patient‐derived kidney organoids compared to the control organoids. The expression of the podocyte development marker *OLFM3* was increased in the patient sample (Figure 3i,j), whereas the expressions of silt diaphragm (*NPHS1*, *NPHS2*, *CD2AP*), WNT signaling pathway (*PLCE1*, *MAGI2*) and cytoskeleton genes (*MYH9*) were decreased (Figure 3i,j). The gene expression changes in the patient were further validated using experimental assays (Figure 3k). Collectively, our results revealed that the podocyte developmental process of the patient‐derived kidney organoid was delayed, and the podocyte structure of the patient‐derived kidney organoid was defective.

### 2.4 Dysregulated Cellular Processes in the Podocytes of Implanted Kidney Organoids Resulting from *WT1* Mutation

**Figure 4:**
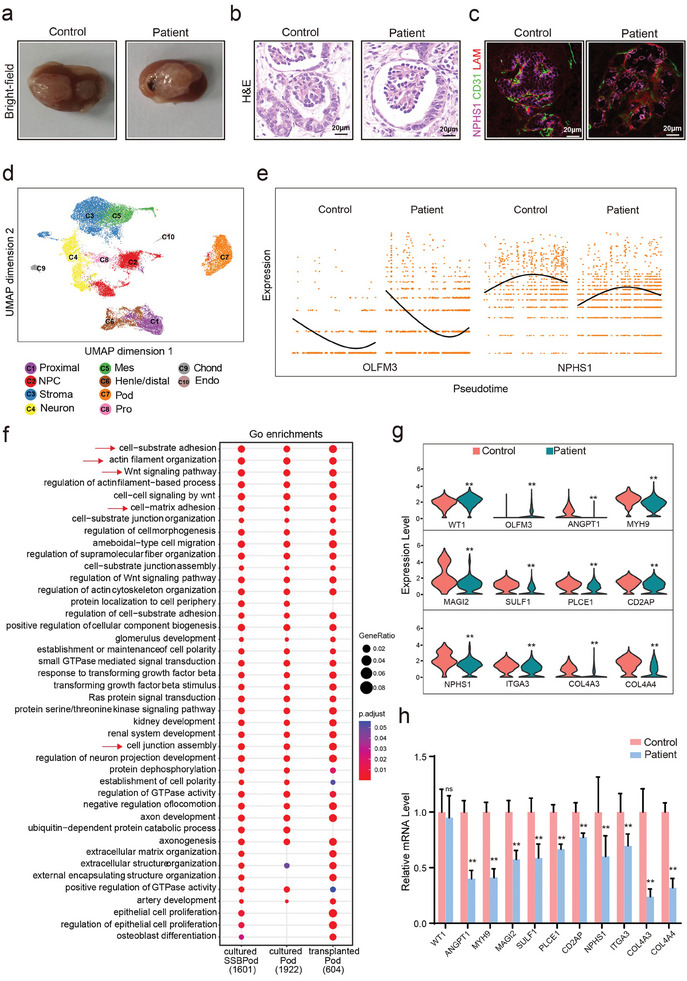
ScRNA‐seq analysis suggests a gene expression profile defect in the three subclusters of podocytes from patient‐derived implanted kidney organoids. a) Bright‐field images of control and patient‐implanted kidney organoids on day 53. b) H&E staining shows that control and patient‐derived implanted kidney organoids contain glomerular structures. c) Confocal immunofluorescence images display that control and patient‐cultured kidney organoids contain nephron compartments, podocytes (NPHS1+), and tubules (ECAD+), Endothelial cells (CD31+). d) uMAP plot depicting ten major cell clusters in implanted kidney organoids of both groups (control group *n* = 2, patient group *n* = 2) on day 53. C1, proximal/proximal tubule; C2, NPC/nephron progenitor cell; C3, stroma; C4, neuron; C5, mes/mesangial cells; C6, distal tubule/loop of Henle; C7, pod/podocytes; C8, pro/proliferating cells; C9, chond/chondrocyte; C10, Endo/endothelial cells. e) Expression of OLFM3 and NPHS1 genes across the pseudotime trajectory colored by cell type. f) First column: GO functional enrichment analysis with differentially expressed genes in the SSBpod clusters in the cultured kidney organoid. Second column: GO functional enrichment analysis with differentially expressed genes in the podocyte clusters in the cultured kidney organoid. Third column: GO functional enrichment analysis with differentially expressed genes in the podocyte clusters in the implanted kidney organoid. The log‐transformed *p*‐value was used to measure significance, and the significance cutoff was set at *p* < 0.05. red arrow: signaling pathways related to kidney development and podocyte structure and function. g) Distribution of scRNA‐seq gene expression of the *WT1*, *ANGPT1*, *MYH9*, *MAGI2*, *SULF1*, *PLCE1*, *CD2AP*, *NPHS1*, *ITGA3*, *COL4A3* and *COL4A4* genes in the SSBpod and podocyte clusters. Data are presented as the mean ± SEM, *p < 0.05 and **p < 0.01 between groups. h) Relative mRNA levels of the *WT1*, *ANGPT1*, *MYH9*, *MAGI2*, *SULF1*, *PLCE1*, *CD2AP*, *NPHS1*, *ITGA3*, *COL4A3* and *COL4A4* genes in the control and patient‐implanted kidney organoids (*n* = 6 per group). Data are presented as the mean ± SEM, *p < 0.05 and **p < 0.01 between groups.

Recently, several studies have shown that kidney organoids implanted under the renal capsule are vascularized by endothelial cells.^[^ ^17^ ^]^ The formation of blood vessels within the implanted kidney organoids induced a mature glomerular filtration barrier. To precisely model the mature podocyte phenotype, we next generated implanted kidney organoids by transplanting the organoids into the renal capsule (**Figure** **4**a). Both the control and patient‐derived kidney organoids exhibited more mature glomerular structures. Unlike the cultured kidney organoids, Bowman's capsule and capillary loop were observed in the implanted kidney organoids (Figure 4b; Figure S8a, Supporting Information). Different components of the implanted kidney organoids were also validated by the immunofluorescent‐based localization of NPHS1/WT1/MAFB (staining podocytes), CD31 (staining endothelial cells), and LAM (staining basement membrane) (Figure 4c; Figure S8b, Supporting Information).

We next performed scRNA‐seq on the control (*n* = 2) and patient (*n* = 2) implanted kidney organoids to study the molecular changes (Figure 4d; Figure S8c, Supporting Information). We identified and defined 10 major cell clusters based on their expression of known marker genes (Figure S8d,e, Supporting Information). As kidney organoids matured, the number of SSBPod cells in both the control and patient groups was largely decreased, making it difficult to directly separate an SSBPod cluster from the podocytes (Figure 4d; Figure S8d, Supporting Information). However, when focusing on the SSBPod marker *OLFM3*, we noticed a substantially increased expression in the podocytes of the patient compared to the control sample (Figure 4e; Figure S8d, Supporting Information), indicating that the podocyte developmental process of the patient‐derived kidney organoid was delayed.

Furthermore, we focused on the podocyte cluster to interrogate the molecular signature underlying the podocyte phenotype caused by *WT1* mutation. GO analysis of the podocyte cluster in the implanted kidney organoid further confirmed the implicated role of the *WT1* mutation in podocyte development and structural pathways, such as the WNT signaling pathway, actin filament organization, and cell‐matrix adhesion (Figure 4f, arrow indication). This result is highly reminiscent of the previous result observed in the cultured kidney organoids (Figures 3, 4, arrow indication). Differential gene expression analysis showed defects in the transcriptome of podocytes in the patient‐derived kidney organoids compared to the control organoids. The expression of the podocyte development marker *OLFM3* was increased in the patient sample (Figure 4g), whereas the gene expressions of the markers of vascularized pathway (*ANGPT1*), silt diaphragm (*NPHS1*, *CD2AP*), WNT signaling pathway (*PLCE1*, *MAGI2*, *SULF1*), cytoskeleton genes (*MYH9*) and cell‐matrix adhesion (*ITGA3*, *COL4A3*, *COL4A4*) were decreased (Figure 4g). The gene expression changes in the patient were further validated using experimental assays (Figure 4h). Overall, for the implanted kidney organoids, the patient's podocyte development was delayed and the podocyte structure was defective, which eventually led to glomerular filtration barrier damage.

### 2.5 The Podocyte Phenotype was Rescued by CRISPR/Cas9 Editing of Patient‐Derived Kidney Organoids Carrying the *WT1* Mutation

To corroborate the finding that the c.1306A>G mutation identified in the patient is the cause of the disease, we performed CRISPR/Cas9‐mediated gene editing to generate gene‐corrected iPSC lines (Figure S9a, Supporting Information). The success rate of our gene‐correction method is as high as 70% (see Experimental Section). The repair template included the wild‐type sequence with an A at the 1306 position as well as an additional synonymous G‐to‐A change to prevent continued Cas9‐mediated cleavage after the successful homology‐directed repair (Figure S9b, Supporting Information). We also experimentally validated the identity of the gene‐corrected iPSC lines by staining the iPSC markers OCT3, TRA‐1‐60, and SOX2 (Figure S9c,d, Supporting Information).

**Figure 5:**
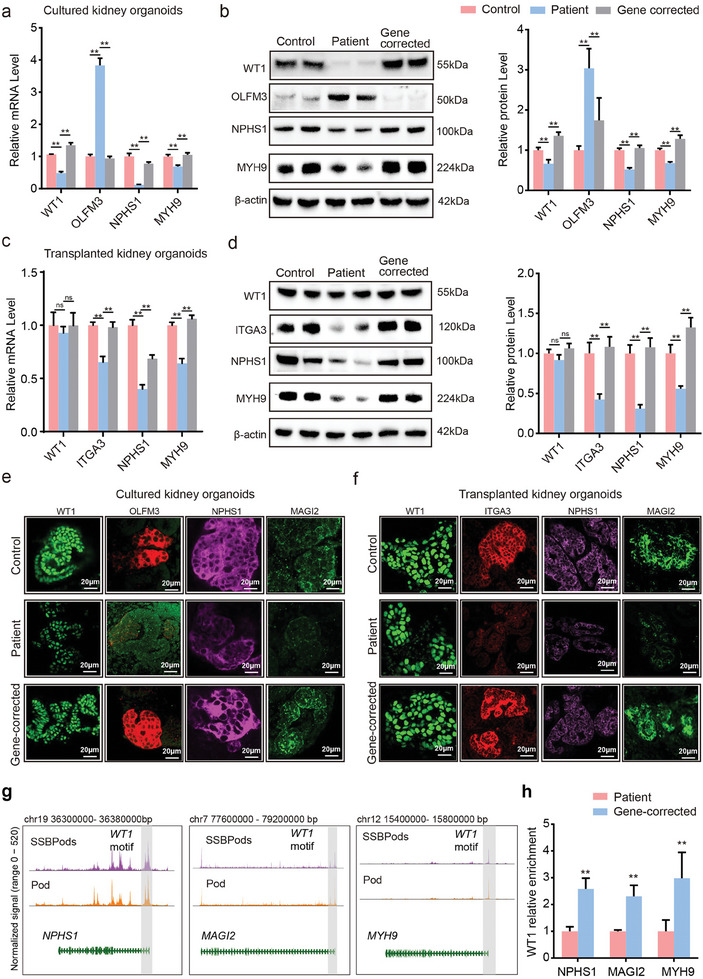
The podocyte phenotype was rescued by CRISPR/Cas9 editing of patient‐derived kidney organoids carrying the WT1 mutation. a) Relative mRNA levels of the WT1, *OLFM3*, *NPHS1*, and *MYH9* genes in the control, patient, and gene‐corrected cultured kidney organoids (*n* = 6 per group). Data are presented as the mean ± SEM, *p < 0.05 and **p < 0.01 between groups. b) Representative Western blot reflecting WT1, OLFM3, NPHS1, and MYH9 protein levels in the control, patient, and gene‐corrected cultured kidney organoids (*n* = 6 per group). Data are presented as the mean ± SEM, *p < 0.05 and **p < 0.01 between groups. c) Relative mRNA levels of the *WT1*, *ITGA3*, *NPHS1*, and *MYH9* genes in the control, patient‐derived, and gene‐corrected implanted kidney organoids (*n* = 6 per group). Data are presented as the mean ± SEM, *p < 0.05 and **p < 0.01 between groups. d) Representative Western blot reflecting WT1, *ITGA3*, NPHS1, and MYH9 protein levels in the control, patient, and gene‐corrected implanted kidney organoids (*n* = 6 per group). Data are presented as the mean ± SEM, *p < 0.05 and **p < 0.01 between groups. e) Immunofluorescence localization of WT1, OLFM3, NPHS1, and MAGI2 in the control, patient‐derived, and gene‐corrected cultured kidney organoids. Scale bars, 20 µm. f) Immunofluorescent localization of WT1, ITGA3, NPHS1, and MAGI2 in the control, patient‐derived, and gene‐corrected implanted kidney organoids. Scale bars, 20 µm. g) Genome tracks of cell type‐resolved aggregate scATAC‐seq data around the NPHS1, MAGI2, and MYH9 gene loci and predicted WT1 binding sites. h) WT1 dynamic binding at three binding sites of the *NPHS1*, *MAGI2*, and *MYH9* genes measured by WT1 direct ChIP‒qPCR from cultured kidney organoids (*n* = 3 per group). Data are presented as the mean ± SEM, *p < 0.05 and **p < 0.01 between groups.

Next, the mRNA and protein levels of the genes in silt diaphragm (*NPHS1/MAGI2*), podocyte development (*OLFM3*), and cytoskeleton (*MYH9*) pathways were clearly changed in the patient‐cultured kidney organoids, which were subsequently rescued in the gene‐corrected cultured kidney organoids (**Figure** **5**a,b). These observations were also confirmed by immunofluorescence experiments (Figure 5e). Furthermore, the mRNA and protein levels of the genes involved in silt diaphragm (*NPHS1/MAGI2*), cell‐matrix adhesion (*ITGA3*), and cytoskeleton (*MYH9*) pathways were clearly decreased in the patient‐derived implanted kidney organoids, which were also readily rescued in the gene‐corrected implanted kidney organoids (Figure 5c,d). Again, we further verified these results using immunofluorescence (Figure 5f).

To analyze WT1‐mediated transcriptional alterations upon the *WT1* c.1306A>G mutation, we first used a ChIP‐PCR assay to confirm the WT1 binding activity at the promoter sites of *NPHS1*, *MYH9*, and *MAGI2*. The motif analysis of scATAC‐seq also predicted that WT1 binds to promoter sites of the three genes (Figure 5g). The binding activity at all of these three gene sites decreased in the patient‐derived kidney organoid and was rescued in the gene‐corrected kidney organoids. These epigenomic changes caused by the WT1 mutation were consistent with the observed gene expression changes (Figure 5h).

We further isolated the glomeruli from mouse kidneys for culturing, and subsequently transfected with lentivirus to induce the mutation in the WT1 gene. The expression levels of ITGA3, MYH9, and NPHS1 proteins in the mutated WT1 group were significantly decreased compared to the control group (Figure S10, Supporting Information), providing a cross‐species validation of the molecular phenotype caused by this patient‐derived WT1 mutation.

Thus, our findings demonstrated that the cultured and implanted kidney organoids nicely model the molecular phenotypes of the podocyte, proving the disease‐causing role of a *WT1* mutation at both the epigenomic and transcriptomic levels. Importantly, these podocyte damage phenotypes can be rescued upon gene correction of the mutation.

## 3 Discussion

In this study, we present a resource elucidating the WT1‐related epigenomic landscape of human podocyte development at single‐cell resolution by generating scATAC‐seq/scRNA‐seq experiments in foetal kidney and kidney organoids. We revealed the functional implications of WT1‐targeted genes, which are crucial for podocyte development and maintenance of podocyte structure. Furthermore, we also confirmed the similarity of kidneys and kidney organoids, the latter of which is a better model for kidney research.

By coupling these dynamic WT1 motif activity maps with WT1‐targeted gene expression at NPC, PTA, SSBpods, and podocytes, we identified several previously WT1‐related functional implications that are important for podocyte development and maintaining podocyte structure. For example, the WNT signaling pathway plays an important role in maintaining the proliferation and differentiation of nephron progenitor cells, as well as regulating the MET pathway. The RHO GTPase cycle, actin filament organization, cell junction assembly, and extracellular matrix organization could regulate and maintain the specific foot‐like structure of podocytes. For the study of transcription factors, ChIP experiments are a relatively direct and suitable method for finding transcription factor binding genes. However, due to the scarcity of foetal kidney samples, we used the scATAC‐seq method for the prediction of transcription factor binding genes. Furthermore, for research at the kidney organoid level, we plan to conduct WT1 ChIP‐seq in the next step to comprehensively verify the transcriptional regulatory role of WT1.

We next used control, patient‐derived, and gene‐corrected kidney organoids to confirm the disease‐causing role of the identified *WT1* mutation. This mutation disrupted highly conserved amino acid residues in the zinc finger regions of the protein. We found that patient‐derived kidney organoids showed delayed podocyte development and damaged podocyte structure, which dynamically modeled the molecular and cellular phenotype of the nephrotic syndrome patient.

The heterozygous missense mutation in *WT1* located in the zinc finger domain did not result in reduced WT1 protein expression. Mutations in exons 8 and 9 of *WT1* usually lead to abnormal zinc finger structures, which lead to a variety of inherited kidney diseases, including nephrotic syndrome, Wilms tumor, DDS syndrome, and so on.^[^ ^9^ ^]^ Previous studies showed that the molecular and cellular phenotype induced by *WT1* mutation usually focused on the role of a single gene or events that occurred at a specific development time point in the nonpatient‐specific model.^[^ ^4^ ^]^ However, WT1 plays a role in the whole process of podocyte maturation and affects multiple gene functional units.^[^ ^1^ ^]^ Therefore, to accurately determine the molecular and cellular phenotypes of *WT1* mutations, we should adopt cultured and implanted kidney organoids that dynamically display the molecular and cellular phenotypes in a sequential manner. Combining kidney organoids and scRNA‐seq, we further dynamically characterized the molecular phenotype of nephrotic syndrome induced by *WT1* mutation. GO term gene functional analysis of SSBPod and podocytes in kidney organoids confirmed an implicated role for *WT1* mutation in podocyte development and structural pathways, such as the WNT signaling pathway(*MAGI2*), actin filament organization*(MYH9)* and cell junction assembly(*NPHS1*). Finally, correcting the mutation in the patient iPSCs using CRISPR–Cas9 gene editing rescues the podocyte phenotype of kidney organoids.

Furthermore, kidney organoids as patient‐specific disease models provide a novel method for the rapid validation and characterization of the gene mutation. Many types of research have demonstrated kidney organoids have more advantages and are sufficient to model phenotype and the study mechanism, such as PKD, Fabry disease, nephrotic disease, ciliopathic phenotype, and so on.^[^ ^7^, ^8^, ^18^, ^19^, ^20^ ^]^

In conclusion, WT1 acts as a crucial transcription factor that regulates podocyte development and maintains podocyte structure and function in the process of kidney development and kidney organoid differentiation.

## 4 Experimental Section

### Ethics Statement

Written informed consent was obtained from the patient, and the study was approved by the Human Subjects Committee of Jinling Hospital, Nanjing University(No. 2022DZKY‐099‐01. Clinical evaluation of the family included a full family history, physical examination, and renal pathology when appropriate. The patient presents with typical clinical symptoms of nephrotic syndrome, such as excessive proteinuria and hyperlipidemia (Figure S7d, Supporting Information). Renal pathology reports and slides were reviewed when available for the patient. The WT1 mutation originated from his father, who had a family history of renal inherited disease, which was consistent with familial co‐segregation. His father had received kidney transplant treatment for ESRD, and his grandmother also received dialysis treatment for ESRD.

### Kidney Organoid Induction with iPSCs

iPSCs were induced toward kidney organoid formation following a 25‐day protocol as previously described with minor modifications.^[^ ^12^, ^13^, ^21^ ^]^ First, iPSCs were transformed into primitive streak cells by treating them with 10 µm CHIR99021 for 4 days. Furthermore, primitive streak cells were treated with 200 ng mL^−1^ FGF9, 1 µg mL^−1^ heparin, and 1 µm CHIR99021 for 3 days to induce the nephrogenic intermediate mesoderm. Next, 2D cultures of intermediate mesoderm cells were dissociated into single cells through Accutase treatment. Single cells were cultured in low‐adhesion 6‐well plates to generate 3D kidney organoids. The intermediate mesoderm was exposed to 200 ng/mL FGF9, 1 µg mL^−1^ heparin, 1 µm CHIR99021, 0.1% polyvinyl alcohol (PVA), 0.1% methylcellulose (MC), and 10 µm Rho kinase inhibitor for one day. After removing 10 µm Rho kinase inhibitor, the intermediate mesoderm was exposed to 200 ng/mL FGF9, 1 µg mL^−1^ heparin, 1 µm CHIR99021, 0.1% PVA, and 0.1% MC, resulting in the generation of nephron progenitor cells for four days. After 12 days, all the inducing factors were removed, and the kidney organoids were cultured in a basic medium until day 25.

### Generation of Implanted Kidney Organoids

All animal experiments were conducted following institutional guidelines approved by the Licensing Committee of Zhejiang University (ZJU20210107). M‐NSG mice (NOD‐Prkdcscid Il2rgem1/Smoc mice, 8 weeks old, from the Shanghai Model Organism Center) were anesthetized with isoflurane. The mouse temperature was maintained at 37 °C. All the surgical instruments were sterilized. The backs of the mice were depilated. The skin was removed, and the kidneys were exposed. A small incision was made in the renal capsule. Kidney organoids (25 days old) were implanted under the renal capsule of the kidneys. The kidneys were collected after 28 days of transplantation.

### Reprogramming of Peripheral Blood Mononuclear Cells (PBMCs) into iPSCs

Fresh whole blood was obtained from the patient, and a PBMC isolation kit (Solarbio P8610) was used to isolate highly purified PBMCs from the fresh whole blood. The PBMCs were cultured in H3000 (STEMCELL Technologies) with CC100 (STEMCELL Technologies). PBMCs were then electroporated with OriP/EBNA‐1‐based episomal plasmids (pCXLE‐hOCT3/4, pCXLE‐hSK, and pCXLE‐hUL) expressing the reprogramming factors OCT3/4, SOX2, KLF‐4, L‐MYC and LIN28 using a LONZA 4D‐Nucleofector (Program EO115) with a P3 Primary Cell 4D‐Nucleofector X Kit11. The PBMCs were cultured on the MEF Feeder Cells by using the H3000 with CC100. The iPSC clone emerged after 15 days.

### AAV6/CRISPR–Cas9‐Mediated Genome Editing

The sequence of WT1 single guide RNA sgRNA was 5′ACTTCAAGGACTGTGAACGA3'. The patient iPSCs were treated with a 10 µm ROCK inhibitor (Y‐27632) during 12 h of pre‐electroporation. The cells were harvested with Accutase (Life Technologies). Before electroporation, a nucleofection solution was made by directly mixing 2 µg of a PX458 plasmid per 1 × 10^6^ cells with 20 µL of P3 primary cell solution (Lonza) at room temperature. A total of 500 000 cells were mixed with 2 µg of the PX458 plasmid. Nucleofection was performed with a 4D Nucleofector system (Lonza) using the CA137 program. Immediately after electroporation, the iPSCs were transferred into one well of a 24‐well Geltrex‐coated plate containing 500 µL of mTeSR1 medium with CloneR (StemCell Technology). For generating the gene‐corrected iPSC, a donor vector AAV6 containing a homologous arm was added directly to the cells at 100 K MOI. After 24 h in culture, the medium was changed to mTesR1. Flow cytometry was used to screen and select single GFP‐positive cells, which were placed into 96‐well Geltrex‐coated plates in which 200 µL of mTeSR1 medium with CloneR had been added. For generating the WT1‐KO iPSC, a donor vector AAV6 was not added into the cells.

### Immunofluorescence Staining

An immunofluorescence staining analysis was performed as described previously.^[^ ^21^ ^]^ Briefly, the 2D cells were fixed with 2% paraformaldehyde (PFA) before being blocked with 0.3% Triton X‐100 and 10% bovine serum albumin (BSA) at 4 °C overnight. Cells were incubated with primary antibodies at 4 °C overnight and detected with secondary antibodies. DNA was stained with DAPI. Kidney organoids were fixed with 2% PFA for 20 min and incubated with primary antibodies (Table S1, Supporting Information) overnight at 4 °C. The kidney organoids were then washed five times with PBS and incubated with secondary antibodies with fluorescent labels (Table S2, Supporting Information). After staining, the kidney organoids were dehydrated using a 25%, 50%, 75%, and 100% methanol series for 5 min, followed by clearing using benzyl alcohol and benzyl benzoate (BABB, 1:2 ratio). The clear kidney organoids were mounted on a glass‐bottom dish (NEST Corporation). The stained cells and kidney organoids were observed via confocal microscopy (Nikon).

### Western Blot Assays

The western blot analysis of the targeted proteins in kidney organoids was conducted as described by prior studies.^[^ ^4^ ^]^ The samples were lysed with cold RIPA lysis buffer (Beyotime, Jiangsu, China), followed by centrifuging at 12 000 × g for 15 min at 4 °C. The supernatants were then collected. The protein concentration was determined by the Pierce BCA Protein Assay Kit (Thermo Fisher Scientific, MA, USA). Then, tissue or cell extracts were mixed with 4× loading buffer containing 250 mmol/L Tris‐HCl, 10%SDS, 0.5% bromophenol blue, 50% glycerol, and 7.5% DTT at pH 6.8. Prior to loading on a gel, samples were heated to 99 °C for 10 min. The samples were separated by 10%/7% SDS‐PAGE and subsequently transferred to nitrocellulose membranes (Millipore Corp., Bedford, MA). The membranes were incubated with blocking buffer (Tris‐buffered saline containing 0.1% Tween‐20 and 5% skimmed milk powder) for 2 h at room temperature and then incubated with primary antibodies at 4 °C overnight (Table S1, Supporting Information). After incubation with a secondary horseradish peroxidase‐conjugated IgG for 1 h at room temperature (Table S2, Supporting Information), immunoblots were visualized using the enhanced chemiluminescence western blot detection system (Millipore). The chemiluminescent signal from the membranes was quantified by a Bio‐red scanner using Image lab software.

### qPCR Assays

Extraction of total RNA of kidney organoids and cells and quantitative real‐time PCR were performed as described previously.^[^ ^4^ ^]^ For quantitative real‐time RT‐PCR, the total RNA of organoids and cells was extracted by RNA extracted kit (Tiangen). The mRNA was reverse transcribed to generate cDNA by superscript reverse transcriptase (Invitrogen). Quantitative real‐time PCR was carried out using a MiniOpticon Real‐Time PCR Detection System (BioRad, Hercules, CA). The real‐time PCR reaction solution consisted of 2.0 µL diluted cDNA, 0.2 µm of each paired primer, and 2 × PCR Master Mix (TaKaRa, Otsu, Japan). Amplification of the housekeeping genes β‐actin was measured for each sample as an internal control for sample loading and normalization. The primers used are listed in Table S3 (Supporting Information). The temperature range to detect the melting temperature of the PCR product was set from 60 to 95 °C. The specificity of PCR products was examined by melting curve at the end of the amplification and subsequent sequencing. To determine the relative quantitation of gene expression for genes, the comparative Ct (threshold cycle) method with arithmetic formulae (2^−ΔΔCt^) was used.

### ChIP‐qPCR

ChIP‐qPCR assays were conducted by ChIP assay kit (CST). For WT1 ChIP assays, chromatin was immunoprecipitated from 100 cultured kidney organoids (three independent experiments for cultured kidney organoids). Samples were fixed with 1% formaldehyde in PBS for 5 min at room temperature before termination with 0.125 m glycine. Cells were then lysed in a sonication buffer, as described previously.^[^ ^4^ ^]^ Cross‐linked chromatin was sonicated to obtain DNA fragments of 200 to 600 bp. Immunoprecipitations were performed, as described previously. Antibodies used are listed in the Table. DNA was recovered by phenol‐chloroform extraction and ethanol precipitation. The qPCR analyses were performed on immunoprecipitated DNA using specific primers described in Table S3 (Supporting Information). Fold enrichment of ChIP versus immunoglobulin G (IgG) control was calculated as 2((Ct(IgG) – Ct(input)) – (Ct(ChIP) – Ct(input))).

### scRNA‐Seq Tissue Dissociation and Preparation

The kidney organoids were stored in GEXSCOPETM tissue preservation solution (Singleron) and transported to the Singleron laboratory on ice as soon as possible. The kidney organoids were washed with Hanks’ balanced salt solution (HBSS) 3 times and minced into 1–2 mm pieces. Then, the kidney organoids were digested with 2 mL of GEXSCOPETM tissue dissociation solution (Singleron) at 37 °C for 15 min in a 15‐mL centrifuge tube with sustained agitation. After digestion, 40‐micron sterile strainers were used to filter the samples, and then, the samples were centrifuged at 1000 rpm for 5 min. Then, the supernatant was discarded, and the sediment was resuspended in 1 mL of PBS (HyClone). To remove the red blood cells, 2 mL of GEXSCOPETM red blood cell lysis buffer (Singleron) was added and incubated at 25 °C for 10 min. The solution was then centrifuged at 500 × g for 5 min and suspended in PBS. The sample was stained with trypan blue (Sigma) and evaluated by microscopy.

### Single‐Cell RNA Sequencing (scRNA‐seq) and Statistical Analyses

Single‐cell suspensions were converted to barcoded scRNA‐seq libraries with a Chromium Single Cell 3′ Library, Gel Bead & Multiplex Kit (10x Genomics), following the manufacturer's instructions. Briefly, cells were partitioned with gel beads in emulsion in the Chromium Controller instrument for cell lysis and barcoded reverse transcription of RNA. The libraries were prepared using 10× Genomics library kits and sequenced on an Illumina HiSeq X with 150‐bp paired‐end reads. The raw reads were processed to generate gene expression profiles using an internal pipeline. Briefly, after filtering one read without poly T tails, the cell barcode and UMI were extracted. Adapters and poly A tails were trimmed (fastp V1) before aligning the second read to GRCh38 with ensemble version 92 gene annotation (fastp 2.5.3a and featureCounts 1.6.2). Reads with the same cell barcode, UMI, and gene were grouped together to calculate the number of UMIs per gene per cell. UMI count tables for each cellular barcode were used for further analysis. Cell type identification and clustering analysis were performed with the Seurat program (http://satijalab.org/seurat/, R package, v.3.0.1) using the RNA sequencing data. UMI count tables were loaded into R with the read.table function. Then, the parameter resolution was set to 1.2 for the FindClusters function and performed a clustering analysis. Differentially expressed genes (DEGs) between different samples or consecutive clusters were identified with the function FindMarkers. Gene Ontology (GO) function enrichment analysis was performed on the gene set using clusterProfiler software to find biological functions or pathways that were significantly associated with specifically expressed genes. Compared Gene Ontology (GO) function enrichment analysis was performed by compareCluster(). First, FindMarkers() function was used to find the genes that are different between the control and patient SSBpod/podocyte in the cultured kidney organoid. Second, FindMarkers() function was used to find the genes that are different between the control and patient podocyte in the implanted kidney organoid. Third, the list() containing different gene sets of the above three groups was created. Finally, compareCluster() function was used to do GO enrichment analysis of different gene sets of the above three groups together. The pseudotime trajectory was performed on the gene set using Monocle3 software.

### scATAC‐Seq Tissue Dissociation and Preparation

Single‐cell suspension preparation was described in tissue processing for scRNA‐seq. The single‐cell suspension was centrifuged to remove the supernatant at 300rcf, 4 °C, 5 min. Cell pellets were re‐suspended and mixed with 100 µL lysis buffer (Tris‐HCl (pH 7.4): 10 mm, NaCl: 10 mm, MgCl_2_: 3 mm, Tween‐20: 0.1%, Nonidet P40 Substitute: 0.1%, Digitonin: 0.01%, BSA: 1%, right amount nuclease‐free water), which was diluted five times by lysis dilution buffer (Tris‐HCl (pH 7.4): 10 mm, NaCl: 10 mm, MgCl_2_: 3 mm, BSA: 1%, right amount nuclease‐free water)) before use and then incubated on ice (3–5 min). After that, the mixture was washed with washing buffer (Tris‐HCl (pH 7.4): 10 mm, NaCl: 10 mm, MgCl2: 3 mm, Tween‐20: 0.1%, BSA: 1%, right amount nuclease‐free water) and centrifuged to remove the supernatant at 500rcf, 5 min. The pellet was then resuspended by diluted nuclei buffer (10× Genomics). The nuclei concentration was calculated and the nuclei solution was further used for library construction. scATAC‐Seq library was prepared following the 10× Genomics single‐cell ATAC‐Seq solution using a protocol supplied by the manufacturer. scATAC‐Seq libraries were sequenced using PE150 sequencing on an Illumina NovaSeq platform.

### scATAC Processing and Clustering

Raw sequencing data were converted to fastq format using “cellranger‐atac mkfastq” (10× Genomics,v.2.1.0). scATAC‐seq reads were aligned to the GRCh38 (hg38) reference genome and quantified using “cellranger‐atac count” (10× Genomics, v.2.1.0). Low‐quality cells were filtered out with less than 1000 sequencing fragments or TSS enrichment less than 4. Bin regions overlapped with ENCODE Blacklist regions were excluded from downstream analysis. Next, the iterative latent semantic indexing (LSI) approach (Granja et al., 2019) was used to reduce the dimensionality of the sparse insertion counts matrix from many thousands to tens or hundreds. Canonical correlation analysis (CCA) was applied to match scRNA and scATAC data. Clustering was performed using the addClusters() function in Signac. Then Uniform Manifold Approximation and Projection (UMAP) or t‐distributed stochastic neighbor embedding (t‐SNE) was used to visualize the data.

### Integration of scRNA and scATAC Data

scRNA data were used as a reference dataset to train the classifier and assign a celltype to each scATAC cell. FindTransferAnchors() function from the Seurat package was used to align data across two datasets. Finally, for each cell in the scATAC‐seq data, this integration process found the cell in the scRNA‐seq data that looks most similar and assigns the gene expression data from that scRNA‐seq cell to the scATAC‐seq cell.

### Peak Calling

MACS2 perform peak calling was used based on the aggregated insertion sites from all cells of each celltype. A consensus set of peaks uniform‐length non‐overlapping peaks was obtained by selecting the peak with the highest score from each set of overlapping peaks. In brief, peaks were first ranked by their significance. The most significant peak was retained and any peak that directly overlapped with the most significant peak was removed from further analysis. Then, of the remaining peaks, this process was repeated until no more peaks existed.

### scATAC Gene Score/Transcription Factor Activity Analysis

Signac was used to estimate gene expression (also named gene scores) for genes and TF motif activity from scATAC data. Gene scores were calculated using the addGeneScoreMatrix() function with gene score models implemented in Signac. JASPAR2020 motif dataset was used in addMotifAnnotations() function to determine motif presence in the peak set. Then, addDeviationsMatrix() function was used to compute the enrichment of TF activity on a per‐cell basis across all motif annotations based on chromVAR.

### Statistical Analysis

Statistical and bioinformatic analyses were performed using GraphPad Prism v 9.5.0 (GraphPad Software Inc., California, USA) or R v 4.3.1 software (R Core Team, Austria). Data are expressed as means ± SEM. Each experiment was performed with at least three biological replicates. Unless otherwise specified, statistical differences among treatment groups were assessed using unpaired two‐tailed Student's *t*‐tests for two groups. All statistical analyses of experimental *n* numbers and *p* values are described in the figure legends. A level of *p* < 0.05 was considered to indicate significance.

## Conflict of Interest

The authors declare no conflict of interest.

## Supporting information

Supporting Information

## Data Availability

Research data are not shared.

## References

[1] N. D. Hastie , Development 2017, 144, 2862. 28811308 10.1242/dev.153163

[2] L. Dong , S. Pietsch , C. Englert , Kidney Int. 2015, 88, 684. 26154924 10.1038/ki.2015.198 PMC4687464

[3] F. J. Motamedi , D. A. Badro , M. Clarkson , M. Rita Lecca , S. T. Bradford , F. A. Buske , K. Saar , N. Hübner , A. W. Brändli , A. Schedl , Nat. Commun. 2014, 5, 4444. 25031030 10.1038/ncomms5444

[4] S. Ettou , Y. L. Jung , T. Miyoshi , D. Jain , K. Hiratsuka , V. Schumacher , M. E. Taglienti , R. Morizane , P. J. Park , J. A. Kreidberg , Sci. Adv. 2020, 6, eabb5460. 32754639 10.1126/sciadv.abb5460 PMC7380960

[5] M. Ameen , L. Sundaram , M. Shen , A. Banerjee , S. Kundu , S. Nair , A. Shcherbina , M. Gu , K. D. Wilson , A. Varadarajan , N. Vadgama , A. Balsubramani , J. C. Wu , J. M. Engreitz , K. Farh , I. Karakikes , K. C. Wang , T. Quertermous , W. J. Greenleaf , A. Kundaje , Cell 2022, 185, 4937. 36563664 10.1016/j.cell.2022.11.028 PMC10122433

[6] A. N. Combes , L. Zappia , P. X. Er , A. Oshlack , M. H. Little , Genome Med. 2019, 11, 3. 30674341 10.1186/s13073-019-0615-0 PMC6345028

[7] S. Tanigawa , M. Islam , S. Sharmin , H. Naganuma , Y. Yoshimura , F. Haque , T. Era , H. Nakazato , K. Nakanishi , T. Sakuma , T. Yamamoto , H. Kurihara , A. Taguchi , R. Nishinakamura , Stem Cell Rep. 2018, 11, 727. 10.1016/j.stemcr.2018.08.003 PMC6135868 30174315

[8] T. Tran , C. J. Song , T. Nguyen , S.‐Y. Cheng , J. A. McMahon , R. Yang , Q. Guo , B. Der , N. O. Lindström , D. C.‐H. Lin , A. P. McMahon , Cell Stem Cell 2022, 29, 1083. 35803227 10.1016/j.stem.2022.06.005 PMC11088748

[9] A. Lehnhardt , C. Karnatz , T. Ahlenstiel‐Grunow , K. Benz , M. R. Benz , K. Budde , A. K. Büscher , T. Fehr , M. Feldkötter , N. Graf , B. Höcker , T. Jungraithmayr , G. Klaus , B. Koehler , M. Konrad , B. Kranz , C. R. Montoya , D. Müller , T. J. Neuhaus , J. Oh , L. Pape , M. Pohl , B. Royer‐Pokora , U. Querfeld , R. Schneppenheim , H. Staude , G. Spartà , K. Timmermann , F. Wilkening , S. Wygoda , et al., Clin. J. Am. Soc. Nephrol. 2015, 10, 825. 25818337 10.2215/CJN.10141014 PMC4422247

[10] S. Domcke , A. J. Hill , R. M. Daza , J. Cao , D. R. O'Day , H. A. Pliner , K. A. Aldinger , D. Pokholok , F. Zhang , J. H. Milbank , M. A. Zager , I. A. Glass , F. J. Steemers , D. Doherty , C. Trapnell , D. A. Cusanovich , J. Shendure , Science 2020, 370. 10.1126/science.aba7612 PMC7785298 33184180

[11] M. Hochane , P. R. van den Berg , X. Fan , N. Bérenger‐Currias , E. Adegeest , M. Bialecka , M. Nieveen , M. Menschaart , S. M. Chuva de Sousa Lopes , S. Semrau , PLoS Biol. 2019, 17, e3000152. 30789893 10.1371/journal.pbio.3000152 PMC6400406

[12] M. Takasato , P. X. Er , H. S. Chiu , M. H. Little , Nat. Protoc. 2016, 11, 1681. 27560173 10.1038/nprot.2016.098 PMC5113819

[13] R. Morizane , J. V. Bonventre , Nat. Protoc. 2017, 12, 195. 28005067 10.1038/nprot.2016.170 PMC5278902

[14] C. Nagano , Y. Takaoka , K. Kamei , R. Hamada , D. Ichikawa , K. Tanaka , Y. Aoto , S. Ishiko , R. Rossanti , N. Sakakibara , E. Okada , T. Horinouchi , T. Yamamura , Y. Tsuji , Y. Noguchi , S. Ishimori , H. Nagase , T. Ninchoji , K. Iijima , K. Nozu , Kidney Int Rep 2021, 6, 2114. 34386660 10.1016/j.ekir.2021.05.009 PMC8343804

[15] H. Hashimoto , X. Zhang , Y. Zheng , G. G. Wilson , X. Cheng , Nucleic Acids Res. 2016, 44, 10165. 27596598 10.1093/nar/gkw766 PMC5137435

[16] M. Jiang , H. Chen , G. Guo , Kidney Dis. 2021, 7, 335. 10.1159/000517130 PMC8443939 34604340

[17] C. W. van den Berg , L. Ritsma , M. C. Avramut , L. E. Wiersma , B. M. van den Berg , D. G. Leuning , E. Lievers , M. Koning , J. M. Vanslambrouck , A. J. Koster , S. E. Howden , M. Takasato , M. H. Little , T. J. Rabelink , Stem Cell Rep. 2018, 10, 751. 10.1016/j.stemcr.2018.01.041 PMC5918682 29503086

[18] S. Karp , M. R. Pollak , B. Subramanian , Micromachines 2022, 13. 10.3390/mi13091384 PMC9506184 36144007

[19] T. A. Forbes , S. E. Howden , K. Lawlor , B. Phipson , J. Maksimovic , L. Hale , S. Wilson , C. Quinlan , G. Ho , K. Holman , B. Bennetts , J. Crawford , P. Trnka , A. Oshlack , C. Patel , A. Mallett , C. Simons , M. H. Little , Am. J. Hum. Genet. 2018, 102, 816. 29706353 10.1016/j.ajhg.2018.03.014 PMC5986969

[20] S. Cui , X. Fang , H. Lee , Y. Jin Shin , E.‐S. Koh , S. Chung , H. S. Park , S. W. Lim , K. I. Lee , J. Y. Lee , C. W. Yang , B. H. Chung , J. Transl. Med. 2023, 21, 138. 36814269 10.1186/s12967-023-03992-0 PMC9948377

[21] S. V. Kumar , P. X. Er , K. T. Lawlor , A. Motazedian , M. Scurr , I. Ghobrial , A. N. Combes , L. Zappia , A. Oshlack , E. G. Stanley , M. H. Little , Development 2019, 146, dev172361. 30846463 10.1242/dev.172361 PMC6432662

