# Supplementary material for: Kidney Organoid Modeling of WT1 Mutations Reveals Key Regulatory Paths Underlying Podocyte Development
Source: Adv Sci (Weinh). 2024 May 29;11(29):2308556. doi: 10.1002/advs.202308556 (PMC11304319; doi:10.1002/advs.202308556)
Supplement: Supplementary file 1 — Supporting Information [file ADVS-11-2308556-s001.docx]

**Supporting Information**

**Kidney organoid modeling of WT1 mutations reveals key regulatory paths underlying podocyte development**

Gang Wang^a,1^, Hangdi Wu^b,1^, Xiuwen Zhai^a,1^, Li Zhang^b,c,e^, Changming Zhang^a^, Chen Cheng^b,c,e^, Xiaodong Xu^a^, Erzhi Gao^a^, Xushen Xiong^b,c,d*^, Jin Zhang^b^,^c,e,f*^, Zhihong Liu^a,b,c*^


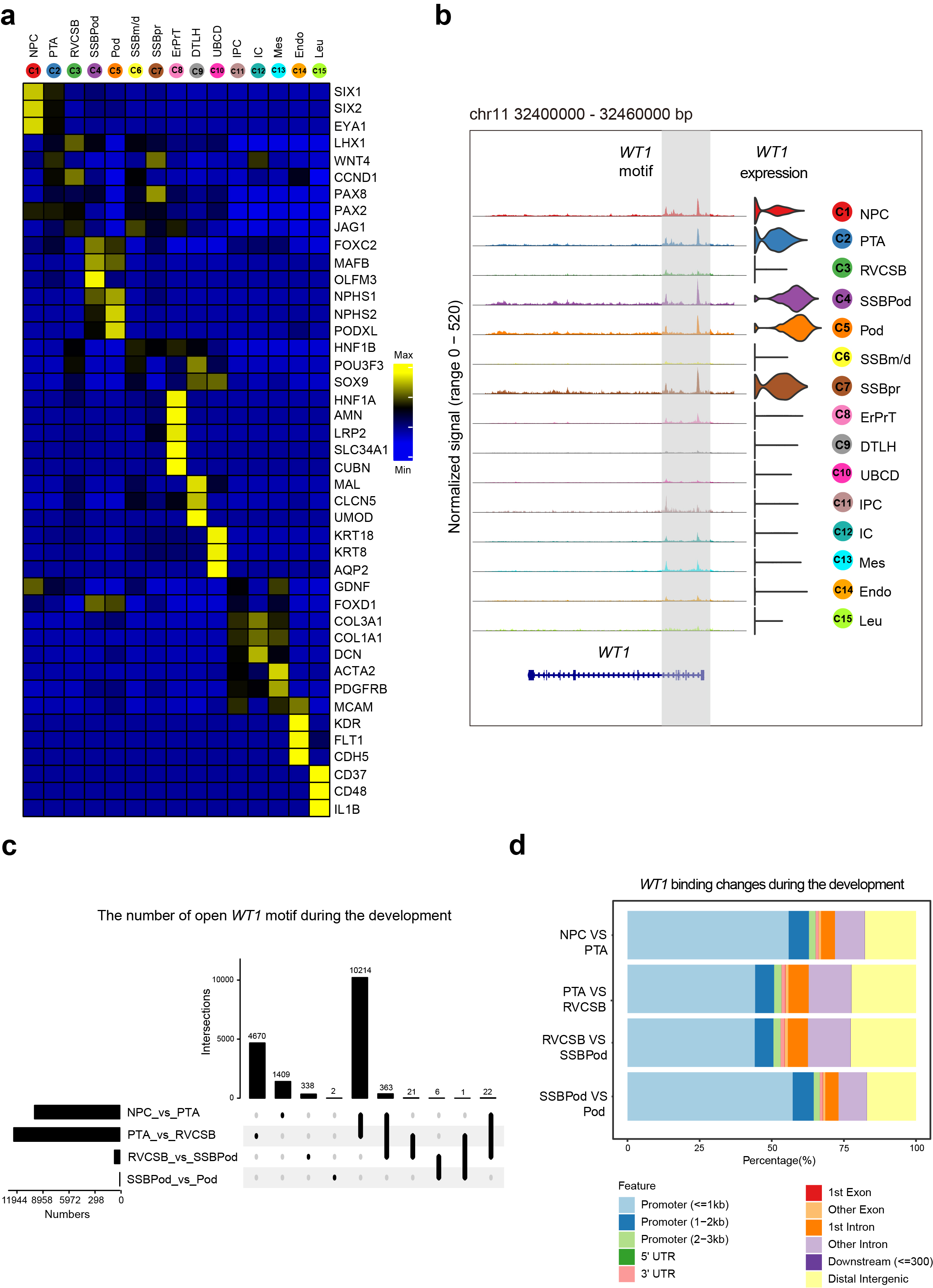


**Figure S1.** (a) Heatmap of literature set gene expression in the 15 identified cell types. Expression was averaged over all cells in a cluster and standardized genewise.

(b) Left column: Genome tracks of cell type-resolved aggregate scATAC-seq data around the *WT1* gene loci. Right column: Distribution of scRNA-seq gene expression of *WT1* across cell type clusters.

(c) Comparison of adjacent developmental stages of genomic WT1 binding sites from NPC to Podocyte. Left bars: sites per time point. Right bars: sites common or unique.

(d) Comparison of adjacent developmental stages of genomic distribution of all WT1 binding sites from NPC to Podocyte.


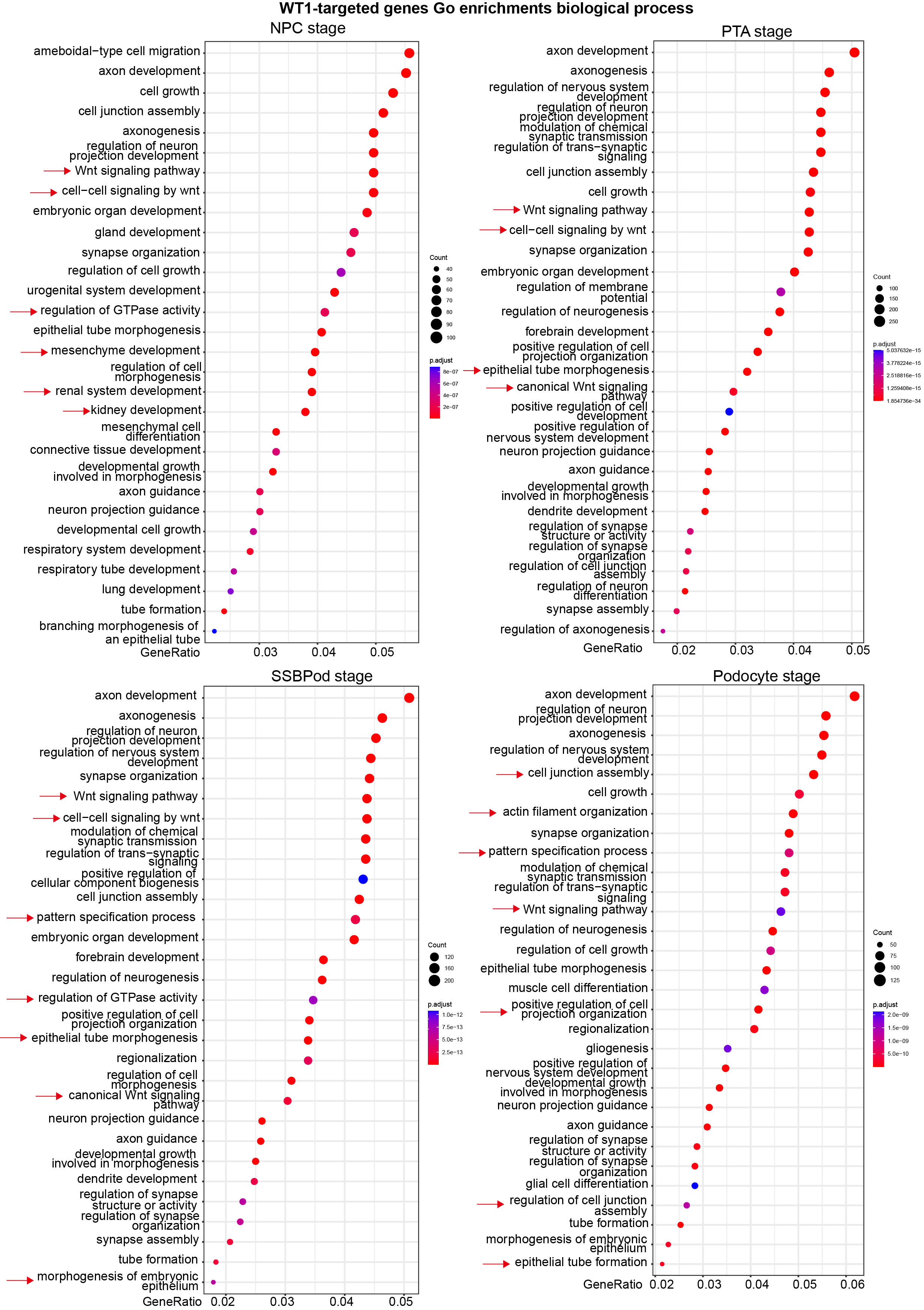


**Figure S2.** Gene Ontology (GO) terms for WT1 binding genes in NPC, PTA, SSBPod and Podocyte stage. red arrow: signaling pathways related to kidney development and podocyte structure and function.


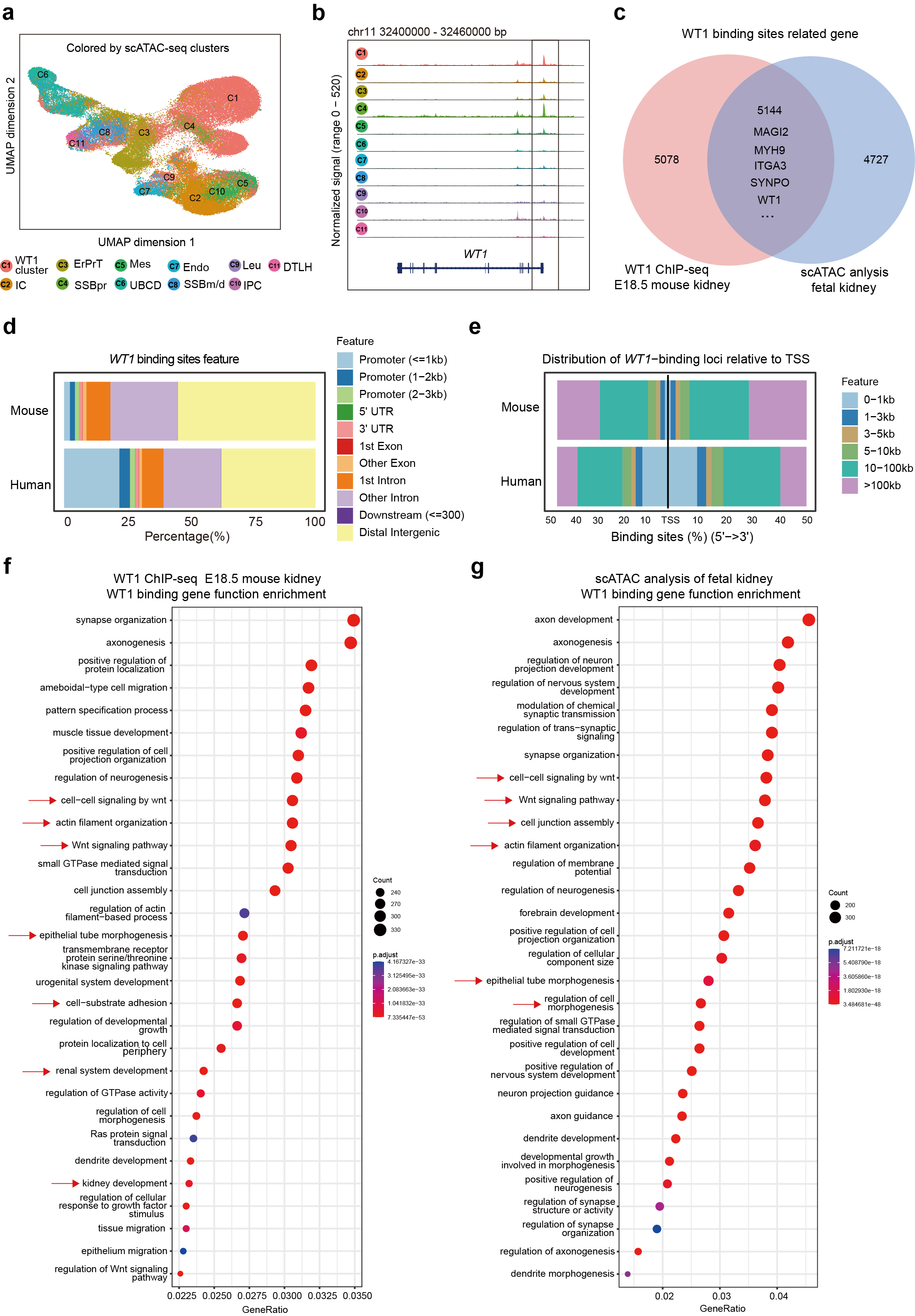


**Figure S3.** (a) UMAPs of scATAC-seq cells coloured by clusters.

C1, WT1_cluster; C2, IC/interstitial cells; C3, ErPrT/early proximal tubule; C4, SSBpr/s-shaped body proximal precursor cells; C5, Mes/mesangial cells; C6, UBCD/ureteric bud/collecting duct; C7, Endo/endothelial cells; C8, SSBm/d/s-shaped body medial/distal; C9, Leu/leukocytes; C10, IPC/interstitial progenitor cells; C11, DTLH/distal tubule/loop of Henle.

(b) Genome tracks of cell type-resolved aggregate scATAC-seq data around the *WT1* gene loci.

(c) WT1-bound genes: pink, WT1 ChIP-seq of E18.5 mouse kidney; blue, scATAC analysis of foetal kidney.

(d&e) Genomic distribution of all WT1 binding sites in both datasets.

(f) GO terms enriched for WT1 binding genes in the WT1 ChIP-seq dataset of E18.5 mouse kidneys. Red/blue shading, P value; circle size, percent of gene set expressed. red arrow: signaling pathways related to kidney development and podocyte structure and function.

(g) Gene Ontology (GO) terms for WT1 binding genes in the scATAC analysis of foetal kidney. red arrow: signaling pathways related to kidney development and podocyte structure and function.


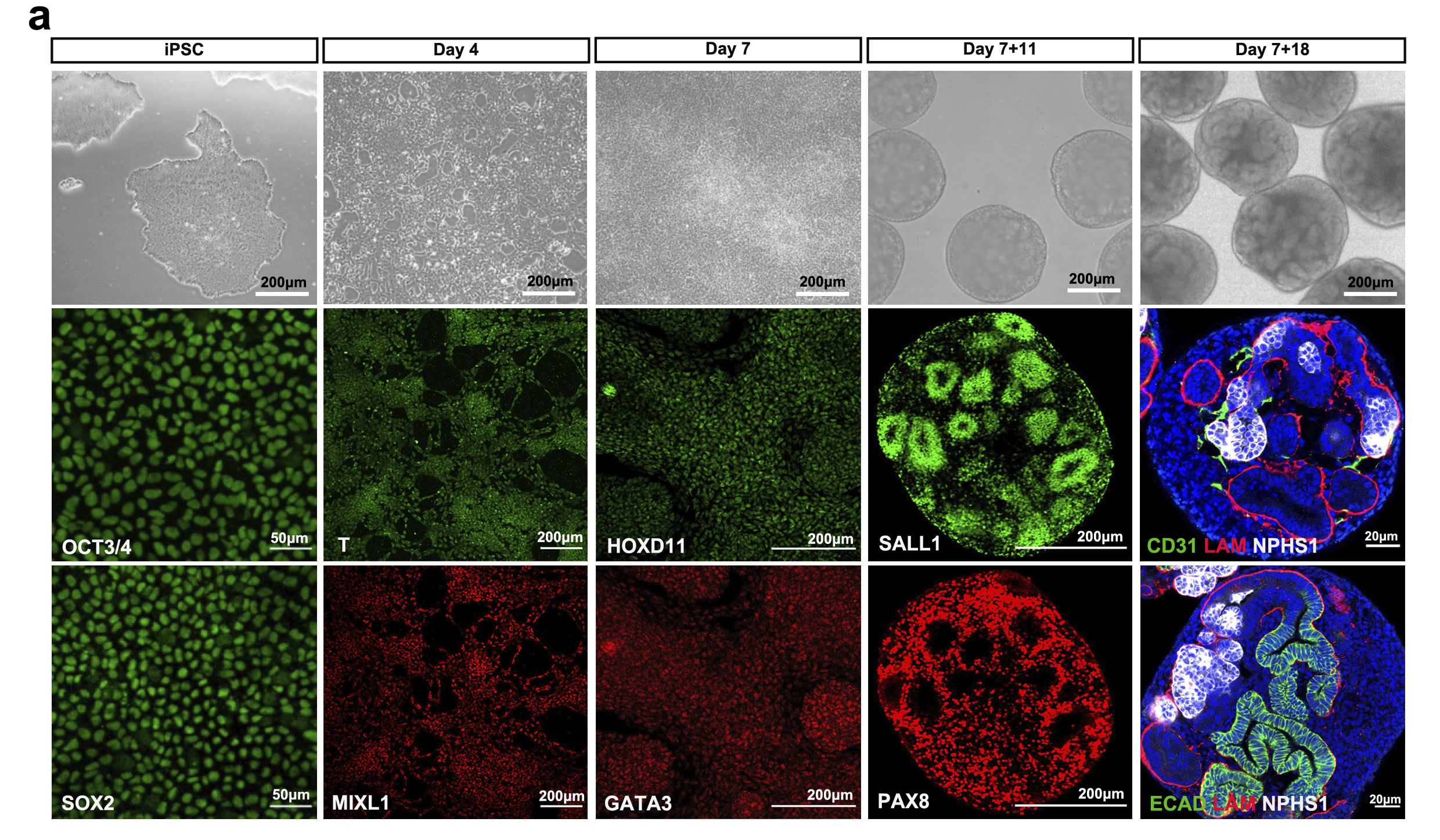


**Figure S4.** Generation of cultured kidney organoids. Up panel: Bright-field image of iPSC, Day4(), Day7(primitive streak), Day7+11(nephron progenitor), Day7+18(kidney organoid). Down panel: confocal immunofluorescence image showing iPSCs (OCT3/4+ and SOX2+), primitive streak (T+ and MIXL1+), intermediate mesoderm (HOXD11+ and GATA3+), nephron progenitor (SALL1+ and PAX8+), endothelial cells (CD31+), podocyte (NPHS1+), basilar membrane (LAM+) and renal tubule (ECAD+).


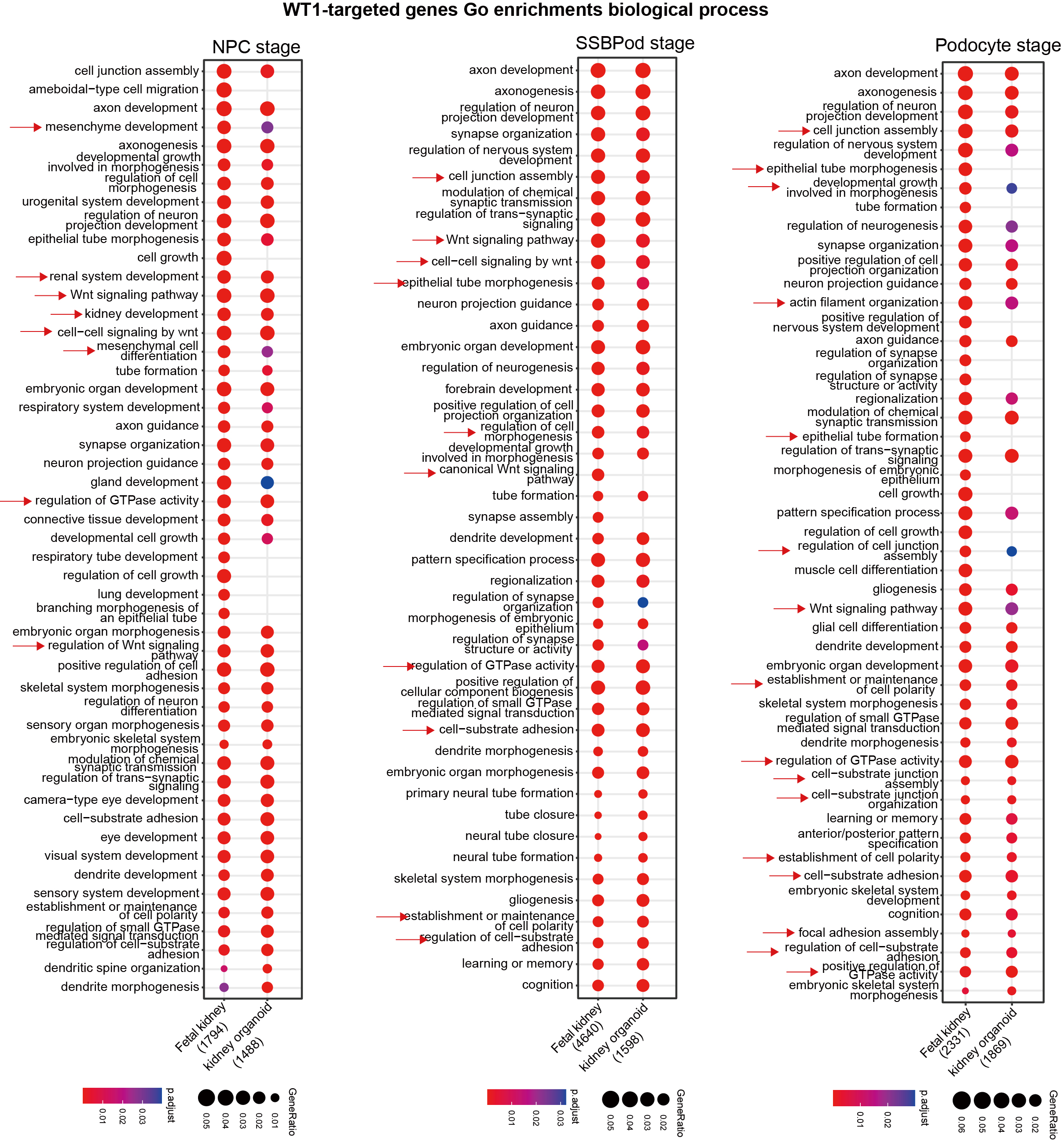


**Figure S5.** Compared GO terms for WT1 binding genes in the NPC, SSBPod and podocyte stage between the foetal kidney and cultured kidney organoid. red arrow: signaling pathways related to kidney development and podocyte structure and function.


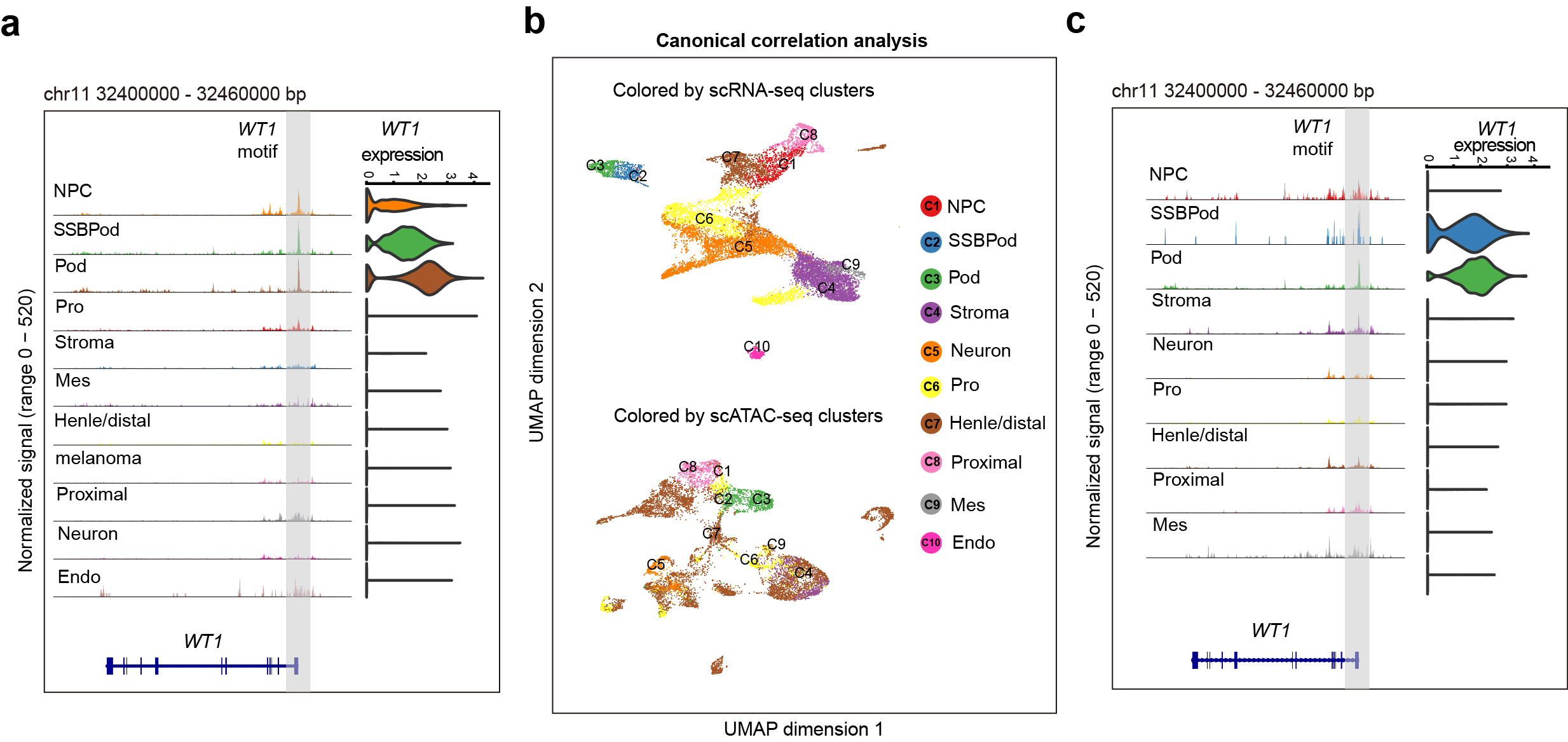


**Figure S6.** (a) Left column: Genome tracks of cell type-resolved aggregate of cultured kidney scATAC-seq data around the *WT1* gene loci. Right column: Distribution of scRNA-seq gene expression of *WT1* across cell type clusters.

(b) UMAPs of scRNA-seq and scATAC-seq of implanted kidney organoid coloured by cluster assignment in their respective data modality and UMAP of scATAC-seq cells highlighted by complementary scRNA-seq clusters.

(c) Left column: Genome tracks of cell type-resolved aggregate of implanted kidney scATAC-seq data around the *WT1* gene loci. Right column: Distribution of scRNA-seq gene expression of *WT1* across cell type clusters.


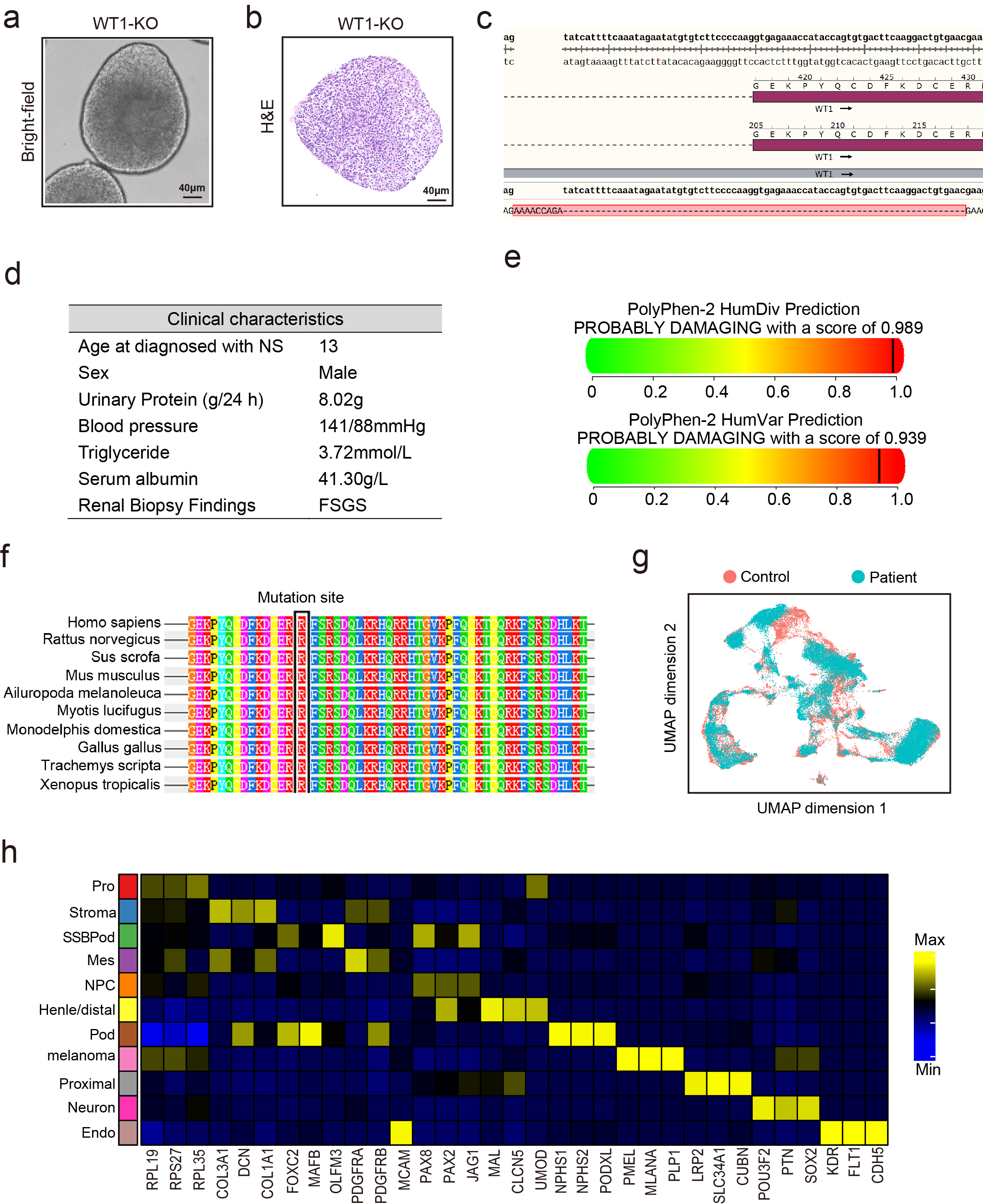


**Figure S7.** (a) Bright-field images of *WT1*-KO cultured kidney organoids on day 25.

(b) H&E staining shows that *WT1*-KO kidney organoids contain no glomerular structures.

(c) Sanger sequencing of exon 8 in *WT1*-KO iPSCs.

(d) Clinical characteristics of patient.

(e) The site is predicted to be damaging by PolyPhen.

(f) Conservation of the amino acid loci across species.

(g) A UMAP plot of combined control and patient-cultured kidney organoid data coloured by sample type.

(h) Heatmap of literature set gene expression in the 11 identified cell types. Expression was averaged over all cells in a cluster and standardized genewise.


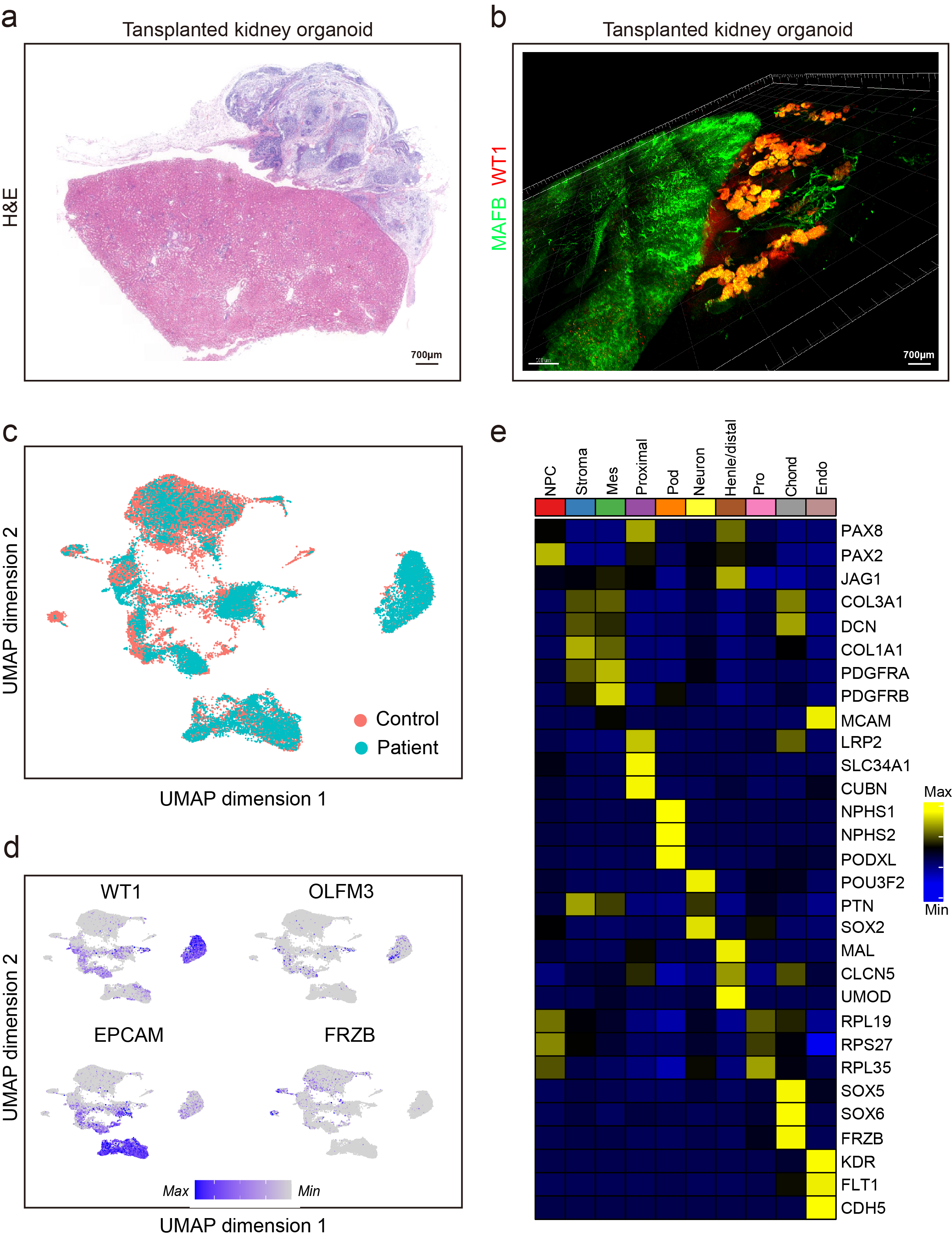


**Figure S8.** (a) H&E staining shows the entire implanted kidney organoids;

(b) Confocal immunofluorescence images display a whole picture of implanted kidney organoids containing podocytes (WT1/MAFB+).

(c) A UMAP plot of combined control and patient-cultured kidney organoid data coloured by sample type.

(d) scRNA-seq of *WT1*, *OLFM3*, *EPCAM* and *FRZB* cells in the clusters.

(e) Heatmap of literature set gene expression in the 10 identified cell types. Expression was averaged over all cells in a cluster and standardized gene wise.


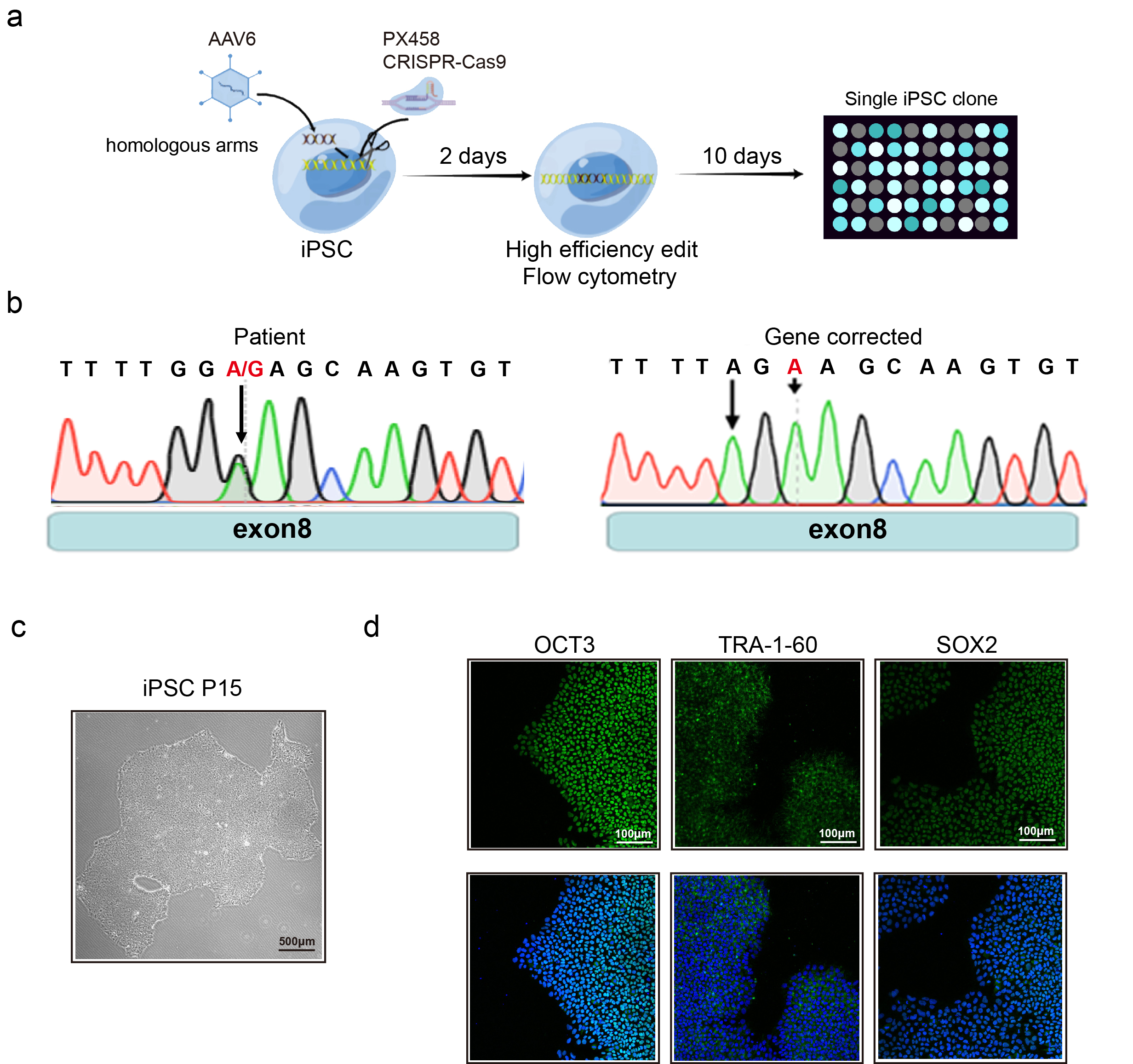


**Figure S9.** (a) Schematic depicting the genome editing process. using CRISPR‒Cas9 plasmid (PX458) and AAV6-mediated donor delivery, followed by flow cytometry analysis after 2 days, allowing genotyping of clones within 10 days.

(b) Sanger sequencing showing that the gene-correction template for the c.1306A>G variant contains the corrected 1 bp substitution (green) and a synonymous upstream 1 bp substitution (red).

(c) The iPSC displayed the classic shape of an iPSC clone.

(d) Immunofluorescence analysis revealed that iPSCs expressed pluripotent markers, such as SOX2, TRA-1-60 and OCT3.


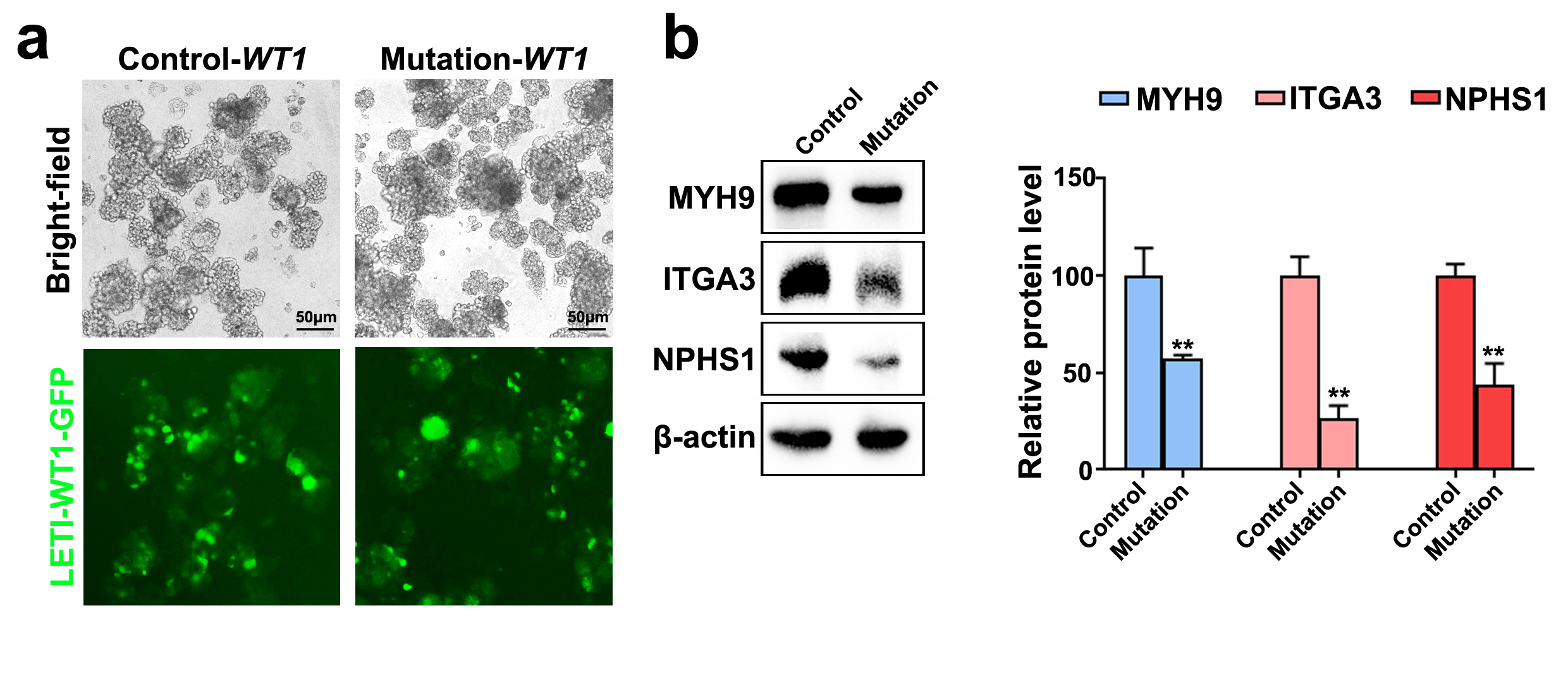


**Figure S10.** (a) Immunofluorescence images display mouse glomerulus transfected by lentivirus containing control/mutation-WT1-GFP. (b) Representative Western blot reflecting MYH9, ITGA3 and NPHS1 protein levels in the control and mutation mouse glomerulus (n = 4 per group). Data are presented as the mean ± SEM, *p < 0.05 and **p < 0.01 between groups.

**Table S1 Primary antibody**

| NPHS1 | R&D Systems | AF4269 | AB_2154851 |
| --- | --- | --- | --- |
| ECAD | BD Biosciences | 610181 | AB_397580 |
| LAM | Abcam | ab77175 | AB_1566369 |
| CD31 | BD Biosciences | 555444 | AB_395837 |
| WT1 | Abcam | ab89901 | AB_2043201 |
| MAFB | SantaCruz Biotechnology | sc-74521 | AB_1125981 |
| OLFM3 | Proteintech | 12596-1-AP | AB_2157228 |
| MYH9 | Proteintech | 60233-1-Ig | AB_2881357 |
| Beta-actin | Proteintech | 66009-1-Ig | AB_2687938 |
| ITGA3 | Proteintech | 66070-1-Ig | AB_11042765 |
| MAGI2 | Thermo | PA5-99245 | AB_2818178 |

**Table S2 Secondary antibody**

| HRP-conjugated Affinipure Goat Anti-Rabbit IgG | Proteintech | SA00001-2 | AB_2722564 |
| --- | --- | --- | --- |
| HRP-conjugated Affinipure Goat Anti-Mouse IgG(H+L) | Proteintech | SA00001-1 | AB_2722565 |
| HRP-conjugated Affinipure Rabbit Anti-Sheep IgG(H+L) | Proteintech | SA00001-16 | SA00001-16 |
| Donkey Anti-Rabbit IgG H&L (Alexa Fluor® 488) | Abcam | ab150073 | AB_2636877 |
| Donkey Anti-Rabbit IgG H&L (Alexa Fluor® 647) | Abcam | ab150075 | AB_2752244 |
| Donkey Anti-Rabbit IgG H&L (Alexa Fluor® 568) | Abcam | ab175470 | AB_2783823 |
| Donkey Anti-Mouse IgG H&L (Alexa Fluor® 488) | Abcam | ab150105 | AB_2732856 |
| Donkey Anti-Mouse IgG H&L (Alexa Fluor® 647) | Abcam | ab150107 | AB_2890037 |
| Donkey Anti-Mouse IgG H&L (Alexa Fluor® 555) | Abcam | ab150110 | AB_2783637 |
| Donkey Anti-Sheep IgG H&L (Alexa Fluor® 647) | Abcam | ab150179 | AB_2884038 |
| Donkey Anti-Sheep IgG H&L (Alexa Fluor® 488) | Abcam | ab150177 | AB_2801320 |

**Table S3 Primer**

| **gDNA primer** | Sequence Primer forward (5’-3’) | Sequence Primer reverse (5’-3’) |
| --- | --- | --- |
| WT1 | CACATGGCTGACTCTCTCATTC | CTAACAAGCTCCAGCGAAGTG |
| **qPCR primer** |  |  |
| WT1 | CACAGCACAGGGTACGAGAG | CAAGAGTCGGGGCTACTCCA |
| OLFM3 | GCCGGATTAGATCCTTCCAAG | GGAGCAACAACTGTGCAAATG |
| MYH9 | CAGCAAGCTGCCGATAAGTAT | CTTGTCGGAAGGCACCCAT |
| MAGI2 | TCCGGCTCAAGTGTGTCAAG | AGGTTGTCACGAATGATTTGCT |
| PLCE1 | GCTTCTTAACACGGGACTTGG | CTTCAAGGGCATTGTGCTCTC |
| CD2AP | GGCATGGGAATGTAGCAAGTC | CCACCAGCCTTCTTCTACCTC |
| NPHS1 | CTGCCTGAAAACCTGACGGT | GACCTGGCACTCATACTCCG |
| NPHS2 | ACCAAATCCTCCGGCTTAGG | ACCAAATCCTCCGGCTTAGG |
| ANGPT1 | AGCGCCGAAGTCCAGAAAAC | AGCGCCGAAGTCCAGAAAAC |
| SULF1 | GATCCCCGAGGTTCAGAGGA | GGTGTAGTCACAAAGGCATTGA |
| ITGA3 | TCAACCTGGATACCCGATTCC | GCTCTGTCTGCCGATGGAG |
| COL4A3 | AGCAAGGGTTGTGTCTGTAAAG | CAGAAAATCCTGGCAATCCACT |
| COL4A4 | AGATAAGGGTCCAACTGGTGT | ACCTTTAACGGCACCTAAAATGA |
| **CHIP-PCR primer** |  |  |
| NPHS1 | CCTGGCCTCCCTTCCTTTT | CTTCCCTCTCTCCCACACTC |
| MAGI2 | AGCAGAGGAAGCAGTGGTG | GCATCCCTACCCTCCTTCC |
| MYH9 | CCTTCCTGCTACACCCACTG | TGTTGAATCCCCACCCATCT |
